# Housing modification to prevent malaria in Uganda: an analysis of costs, willingness to pay, and equity

**DOI:** 10.1186/s12936-025-05757-0

**Published:** 2025-12-24

**Authors:** Katherine Snyman, Walter Ochieng, Sam Gonahasa, Joyce Aber, Agaba Katureebe, Sarah G. Staedke, Moses R. Kamya, Nelli Westercamp, Catherine Pitt

**Affiliations:** 1 Infectious Diseases Research Collaboration (IDRC), Plot 2C Nakasero Hill Road, Kampala, Uganda; 2 Department of Global Health and Development, London School of Hygiene and Tropical Medicine (LSHTM), London, UK; 3 Centers for Disease Control and Prevention, Atlanta, GA USA; 4 Liverpool School of Tropical Medicine (LSTM), Liverpool, UK; 5 Department of Medicine, Makerere University, Kampala, Uganda

**Keywords:** Cost analysis, Housing modification, Screening, Eave tubes, Malaria, Willingness to pay, Equity

## Abstract

**Background:**

Innovative, equitable, and sustainable multisectoral solutions are required to address persistently high global malaria deaths, widespread insecticide and antimalarial resistance, and falling funding for malaria control. Housing modification presents a promising option. Alongside a cluster-randomized control trial in Eastern Region, Uganda, we analysed the costs and households’ willingness to pay (WTP) for two housing modification interventions, screening and eave tubes, focusing on equity and scale-up potential.

**Methods:**

Taking a disaggregated societal perspective, we assessed financial and economic costs of installing two housing modification interventions in approximately 4000 homes (20000 people). We collected WTP data through three cross-sectional household surveys (n = 1500 households each) using modified structured haggling and calculated price elasticity of demand. We used multivariable regressions and concentration indices to analyse how costs and WTP varied by household characteristics. To identify potential financing gaps, we compared WTP to costs and examined variation by household wealth quintiles.

**Results:**

Screening cost a mean of $116 (societal economic costs; 95%CI $112–120) United States Dollars (2022 USD) per house; eave tubes cost $50 (95%CI $48–52). When annualized over 5 years, screening cost $4.22 per person protected per year and eave tubes cost $3.03. Installation cost more in the wealthiest versus poorest quintiles for both screening ($151 vs $69) and eave tubes ($95 vs $31). Over 75% of respondents were willing to pay something for the interventions, but these values represented only a small fraction of the costs, with a higher fraction in the wealthiest vs poorest quintiles (screening: 12% vs 7%; eave tubes: 18% vs 14%).

**Conclusions:**

While housing modification has relatively high upfront costs, its annual cost per person protected is comparable to other malaria interventions. Households, especially the poorest, are unwilling or unable to pay the full cost of housing modifications. Equitable scale-up would require additional financing and/or demand-boosting interventions.

*Trial Registration*: NCT04622241 (clinicaltrials.gov).

**Supplementary Information:**

The online version contains supplementary material available at 10.1186/s12936-025-05757-0.

## Background

Despite some modest reductions since 2015, malaria case and death rates have remained persistently high both globally and in Uganda [1]. The burden of malaria disproportionately affects young children and poor, rural populations [2, 3]. Growing insecticide and drug resistance has rendered existing control tools less effective [4–6], and a widening funding gap between the amount invested and resources needed has limited coverage of available prevention and treatment tools [1]. Poor quality housing, which is most common in low-income households [7], is a known risk factor for malaria infection at both household [8] and community levels [9]. Innovative, equitable, sustainable, and multisectoral solutions are therefore needed to complement existing malaria prevention interventions and address these inequities.

Housing modification is a longstanding complementary approach to existing malaria prevention methods. It is defined as “any structural changes, pre- or post-construction, of a house that prevents the entry of mosquitoes and/or decreases exposure of inhabitants to vectors with the aim of preventing or reducing the transmission of malaria” [10]. One approach is to screen openings, such as eaves, windows, doors, ceilings, and other gaps. Another approach is to install eave tubes, which are PVC tubes fitted with electrostatic mesh inserts treated with insecticides, into a home’s exterior walls [11–13]. Because of substantial gaps in the evidence regarding the durability, feasibility, equity, and affordability of housing modification interventions, the World Health Organization (WHO) currently only conditionally recommends residential house screening as a malaria prevention measure [10].

A Cochrane review of housing modifications for malaria prevention identified seven trials conducted in sub-Saharan Africa since 2009 and found that housing modification reduced malaria parasite prevalence by 32% and anaemia prevalence by 30% [14]. Nine economic evaluations have examined the costs and/or cost-effectiveness of housing modification in sub-Saharan Africa [13, 15–22]. In trial settings, the reported cost of screening ranges from $9 to $142 (United States Dollars (USD) 2022) per house modified [15–19, 22] and from $7 to $32 per person protected from malaria annually [13, 15–17]. These variations reflect differences in home types, intervention scope, price levels, types of resources and costs included in estimates, and cost metrics reported. A trial in Côte d’Ivoire reported that compared to long-lasting insecticidal nets (LLINs) alone, eave tubes plus screening plus LLINs cost a median additional $210 per disability-adjusted life year averted from a societal perspective, with a 74% probability of being cost-effective in that setting [22]. A trial in Zambia found the productivity gains from house screening exceeded the costs, but excluded homes in poor condition, raising equity concerns [17]. Studies in Kenya and Tanzania found that > 90% of respondents wanted their homes screened, and 98% of Kenyan homeowners were willing to pay for screening, but the amounts they were willing to pay were not quantified [18, 21]. In Tanzania, a survey of urban households found that > 80% of respondents had screened their homes, paying $21–30 (USD 2009) per home for the modification [20].

Interest in housing modification is growing, but substantial evidence gaps remain. Funding mechanisms for these interventions remain unclear. Housing modification is unlikely to be affordable within ministry of health budgets in high malaria burden countries, as found in Côte d’Ivoire [23]. Moreover, housing modification does not fit within traditional product distribution channels favoured by international donors [24]. These challenges have spurred calls for innovative financing strategies, including private sector investment [1], which have become more pressing with recent reductions in international aid [25]. The widespread expectations that households may organically adopt and finance modifications assume that households will be willing and able to pay the costs of modifying their own homes. No study, however, has evaluated both the costs and the maximum amount households are willing to pay, or willingness to pay (WTP), for housing modifications or quantified their equity implications.

To address these evidence gaps, we evaluated the costs, affordability, and equity of housing modifications and households’ WTP for them within a three-arm cluster-randomised trial in Eastern Region, Uganda. First, we assessed the economic and financial cost of implementing two interventions, screening and eave tubes, from a disaggregated societal perspective. Second, we examined how the cost per house modified varied by household wealth, type, and size. Third, we analysed households’ WTP for modifications and how it varied by demographic characteristics. We combined this evidence to identify the financing gap, which we define as the difference between the cost of housing modifications and the amount households are willing to pay for them. Finally, we assessed the organic uptake and emergence of a local market for housing modifications outside the study context through a survey of construction workers. Our findings offer insights for policymakers considering whether and how to equitably and sustainably scale up housing modification for malaria prevention in Uganda and elsewhere in sub-Saharan Africa.

## Methods

### Study context

Uganda’s 2022 gross domestic product per capita was $964 USD, and 24% of households engaged in subsistence agriculture [26, 27]. Based on national ($1.77 per day) [28] or international ($3.00) [29] poverty lines, 26% or 60% of the country’s population, respectively, consumes less than the minimum income required to meet basic needs. Poverty rates are two times higher in rural compared to urban areas [28]. Housing construction in Uganda varies in quality of materials and methods of construction, even within the same village. In rural areas, most homes have earth floors (73%), mud walls (66%), and iron sheet roofs (73%) [3].

Malaria is endemic in 95% of Uganda’s districts, accounts for more than half of all outpatient care in health facilities, and is the leading cause of morbidity and mortality, especially among children [30] Uganda’s malaria control plan focuses on provision of LLINs through mass community distribution campaigns every 3–4 years, targeted indoor residual spraying (IRS), intermittent preventive treatment in pregnancy, case management, and surveillance [31]. Core malaria interventions are provided free at the point of access and primarily financed (> 90%) by international partners [32].

We conducted the study in Jinja and Luuka districts in Uganda’s Eastern Region, where malaria transmission, parasite prevalence, and insecticide resistance are high (Figure S2) [33]. Eastern Region, Uganda mirrors many of the socio-economic characteristics found across the country. Jinja serves as a commercial and industrial hub, with a growing population engaged in various economic activities [34]. However, much of the surrounding region, including Luuka District, remains predominantly rural, with a substantial proportion of the population dependent on subsistence agriculture. Relative to the whole country, our study population is somewhat poorer (Table 1).

**Table 1:** Description of study population characteristics by intervention arm

Variable		Screening arm n = 1029	Eave tubes arm n = 1025
Head of household characteristics			
Education level	Missing	2 (0%)	5 (0%)
	None	264 (26%)	209 (20%)
	Primary	485 (47%)	492 (48%)
	Secondary	246 (24%)	295 (29%)
	Higher	32 (3%)	23 (2%)
Age	Mean	46.1	45.2
Sex	Missing	2 (0%)	5 (0%)
	Male	753 (73%)	764 (75%)
	Female	274 (27%)	255 (25%)
Household characteristics			
Study wealth quintile	Poorest Q1	190 (18%)	225 (22%)
	Q2	221 (21%)	211 (21%)
	Q3	215 (21%)	193 (19%)
	Q4	220 (21%)	200 (20%)
	Wealthiest Q5	183 (18%)	196 (19%)
National wealth quintile	Poorest Q1	148 (14%)	178 (17%)
	Q2	301 (29%)	281 (27%)
	Q3	401 (39%)	361 (35%)
	Q4	147 (14%)	154 (15%)
	Wealthiest Q5	32 (3%)	51 (5%)
Household size (number of residents)	Mean	5.53	5.56
Type of house	Traditional	658 (64%)	636 (62%)
	Modern	371 (36%)	389 (38%)
House perimeter^a^	Mean meters	28.97	27.54
House area^a^	Mean meters^2^	65.88	57.98
Number of windows screened	Mean	3.34	N/A
Number of eave tubes installed	Mean	N/A	9.18

The study installed screening in 2084 homes and installed eave tubes in 1986 homes. A subset of these homes had both demographic and resource use data collected (screening = 1029; eave tubes = 1025 homes)

^**a**^A smaller subset of homes had data collected on household perimeter and area (screening = 920 homes; eave tubes = 903 homes)

### Study design

**Fig. 1:**
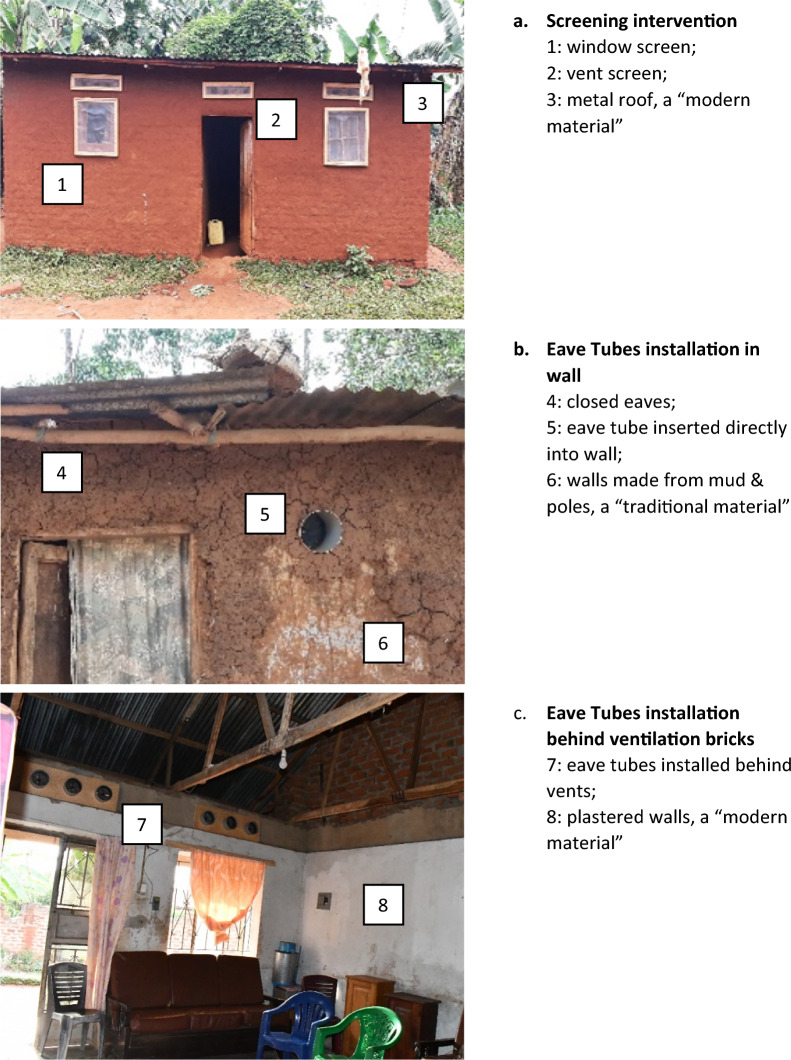
Illustration of interventions: Screening and eave tubes

We report on the economic aspects of the Uganda Housing Modification Study, a two-phase study to evaluate housing modification as a malaria prevention tool. Details of the pilot phase are presented elsewhere [35]. The trial phase, a cluster-randomised control trial, assessed two interventions (screening and eave tubes) relative to one control arm, with 20 clusters—approximately 2000 homes and 10000 people—per arm (60 clusters total). We modified 2084 homes in the screening arm and 1986 home in the eave tubes arm, as detailed elsewhere [36]. We defined “modern” homes as having closed eaves and roofs, walls, and floors made from processed materials; all other homes were defined as “traditional” (Fig. 1).

We conceptualize inequity in housing modifications as unfair access due to unaffordability, particularly for poorer households. We examine the economic burdens these interventions impose, considering both absolute and relative costs compared to household consumption and WTP. In the context of a donor-funded initiative, we assess equity by investigating whether wealthier households receive a disproportionate share of resources. Additionally, we explore variation in burden and allocation by head of household characteristics such as age, sex, and education.

### Interventions

In March 2022, to fill gaps in LLIN coverage following mass LLIN distribution (March–November 2021), research assistants distributed piperonyl-butoxide (PBO) LLINs (n = 8866) at health centres and door-to-door, aiming for universal coverage (one LLIN per two household residents) in all study areas. PBO LLINs were distributed due to high levels of pyrethroid resistance in the study districts.

From December 2021 to April 2022, teams of locally recruited and trained construction workers modified the homes of consenting households in the intervention arms. In villages randomized to the screening arm, the study team installed screens on all open eaves, ventilation bricks/openings, and windows for all households (Fig. 1). The process involved visiting each home to measure openings, building custom frames with mesh at the project workshop, and returning to install the screens. Any gaps in walls, including around door frames, were sealed with cement or mud. Doors were not screened due to maintenance challenges and the common practice of leaving doors open until bedtime [37].

In the eave tubes arm, the study team offered installation of In2Care^®^ EaveTubes in the outer wall of the rooms routinely used by household members. These tubes funnel human odours outwards, attracting malaria mosquitoes, which become trapped by netting impregnated with a lethal dose of deltamethrin insecticide. In a single visit, workers measured the walls and installed eave tubes at intervals of 1.5 m–2.3 m, approximately 20 cm below the roof. Installation methods varied based on the home’s construction and comprised: (1) attaching the tubes to a wooden board behind an existing ventilation brick, (2) drilling into the wall with a specialized drill, or (3) chiselling and hammering to insert the tubes into the wall. Any gaps around the eave tubes were sealed with cement or mud. Eave tube inserts require re-treatment every 12 months [38].

### Data collection

#### Provider costs

We prospectively collected data on costs of housing modification. We collected resource use and cost data on all modification activities review of daily transport logs, daily worker logs, and project procurement, budget, and expenditure records. In addition, study staff directly observed the resources used per output (e.g., number of windows/vents screened) for each house, which allowed for household-level cost estimates.

### Household surveys

We conducted three panel community household surveys (pre-installation, and at 12 and 18 months post-installation) and two cohort household surveys (baseline and end of follow-up) (Fig. S1). For the community surveys, households were randomly sampled in each of the 60 clusters (with resampling across rounds) until 25 households with at least one child aged 6 months to 14 years were enrolled, totalling 1500 households. The cohort surveys included all households with children enrolled in the cohort, which consisted of children under 5 years from 25 randomly selected households per cluster (500 households per arm, 1500 total) who were followed for 1 year.

All surveys included questions about characteristics of households and residents, asset ownership, ownership and use of LLINs, and WTP for housing modification. Using a modified structured haggling technique, research assistants asked the head of the household how much they were willing to pay for installation of screening and eave tubes and for retreatment for eave tubes, regardless of which intervention their cluster was randomized to receive [39] (Fig. S4). We used the intervention costs from the pilot study to inform the initial bids. We asked participants to rank interventions in order of preference and then asked WTP questions about interventions from least to most preferred. Questions about households’ contributions to the modifications (donated time, space, or materials) were only included on the community 12-month survey.

### Construction worker survey

To gauge organic uptake and emergence of a local market for screening, we surveyed construction workers (n = 115; 88% of those hired for the modification activities) in November 2022, nine months after installation (Fig. S3). The survey explored whether the workers had installed screening in homes outside the study setting, the prices they charged, what they would hypothetically charge for future modifications, and the availability of local materials.

### Data analysis

We analysed data using Microsoft Excel and STATA 14 (StataCorp, Texas, USA). Internationally tradable goods were converted using annual exchange rates from the purchase year and then inflated to 2022 USD using gross domestic product (GDP) deflators. Other goods and services were inflated to 2022 Ugandan Shillings (UGX) using GDP deflators and then converted into USD (3691 UGX = 1 USD) [40–42]. All costs are reported 2022 USD unless stated otherwise.

### Cost analysis

We analysed the costs of implementing the interventions from a disaggregated societal perspective, presenting results separately from the provider (project), household, and combined societal perspectives. We estimated financial costs, which reflect resources that are paid for, and economic costs, which reflect the opportunity cost of resources used, including donated items or time. Capital costs were valued in the financial analysis by the price paid for them and in the economic analysis by subtracting their residual value at the end of the study from their starting value. Research costs were excluded. Donated services, goods, and space from households were valued using market rates, while donated time was valued using mean monthly rural household consumption (545,747 UGX) and the average number of adults per household (n = 2.5), yielding a daily rate of 9223 UGX ($2.69) [26]. Informal discussions with local study staff informed values for donated meals ($0.82) and storage ($0.66 per day). We applied a top-down gross costing approach, estimating the total cost of resources and diving by associated outputs [43]. The mean cost per house was calculated by dividing total resource costs by the number of houses modified. To assess cost variation across households, costs were allocated by unit outputs (e.g., number of screens, and eave tubes installed per home). We present the mean cost per house modified to reflect the total cost burden and enable straightforward cost extrapolation, making it more suitable for economic evaluations and policy decisions.

Economic costs are presented, unless otherwise specified. We categorized all costs as capital or recurrent and by category (materials and supplies, labour, tools and equipment, local transportation, international transportation and import fees, storage and local workshop, supervision, training, and community sensitization). As well as the cost per house modified, we estimated the annual cost per person protected using the observed mean number of household members (n = 5.5). We annualized the upfront costs of housing modification assuming a 3% discount rate and alternative intervention lifespans of 5 and 15 years, given the unknown and inconsistent lifespans reported in previous studies. For eave tubes, we added annual retreatment cost estimates from a similar trial ($6.65 per house) [22].

We conducted univariable sensitivity analysis separately for each intervention to determine the most influential parameters in the annual cost per person protected (tornado diagrams in supplementary materials). We used existing literature and price variations recorded during the study to define the range over which input parameters were varied.

For equity analyses, we generated a socio-demographic dataset by combining all five household surveys (3609 households; 60% of all households in the study). We constructed a study wealth index using principal components analysis [44, 45], and used the EquityTool to assign the households to national wealth quintiles and their associated mean per capita consumption expenditure [46]. We then generated a dataset of 2054 homes (screening: 1029 homes; eave tubes: 1025 homes) with both housing modification cost data and socio-demographic information. A subset of the latter (1832 homes) also had data on household perimeter and area (screening: 930 homes; eave tubes: 902 homes). We ensured that the various data subsets were representative of the overall dataset.

To understand the equity and relative affordability of housing modification to households, we assessed how modification costs varied by physical (house size and type) and sociodemographic equity-relevant variables (wealth, head of household age, sex, and education level). We compared the cost per house modified with consumption expenditure by national quintile [29] and examined variation in cost per house modified using univariable regression.

We conducted a multivariable analysis to explore equity-relevant factors associated with variations in the cost of housing modifications, incorporating variables selected *a priori* [47]*.* Outliers were excluded to ensure data quality, and key covariates such as household head’s education level (binary), age (categorical), and household wealth (categorical) were constructed. Costs were log-transformed to stabilize variance, and the coefficients from the regression model were interpreted as percentage changes in the dependent variable. We translated the log-transformed coefficients back into raw cost values, using a smearing estimator [48]. To identify a parsimonious model, we started with all independent variables and employed stepwise backward elimination using likelihood ratio tests, retaining variables with a selection parameter of 0.05 [49]. Concentration curves and indices were produced in STATA using the *conindex* command with the study wealth index as the ranking variable. A concentration curve depicts the distribution of a health outcome across socioeconomic groups [50, 51].

### Willingness to pay

To evaluate potential market demand, we estimated the mean maximum WTP for intervention installation and eave tube re-treatment, disaggregated by wealth. Estimates were calculated including and excluding responses indicating zero WTP. We used a difference-of-means test to compare WTP across different survey time points and interventions. We assessed variation in WTP using univariable and multivariable regression analyses across all timepoints. We categorized household head’s age and sex as binary variables and classified education level and household wealth as categorical variables. Our dependent variable had a highly skewed inflated distribution, so we used a two-part model [52]. Again, we used backwards elimination with a selection parameter of 0.05 to identify the most parsimonious model [49]. We performed goodness-of-fit tests to ensure correct model specification [53]. We generated point elasticities of demand, which quantify the percentage change in quantity demanded in response to a one percent change in price, for each intervention, overall and by household wealth and intervention arm by regressing log (quantity demanded) on log (price), where the coefficient on price represents the elasticity.

### Financing gap

To identify the potential financing gap for private uptake of housing modifications, we compared the cost of each home modified with that household’s WTP for modification. We calculated the proportion of households willing to pay the full cost of modifying their homes, and the mean proportion of modification costs that households were willing to pay, both in total and by wealth quintile. Data were available for 47% (979/2042) of homes that were screened and 50% (985/1964) of homes that received eave tubes.

### Organic uptake outside study

To understand local market dynamics and validate our cost estimates, we analysed the number of homes modified by study-trained construction workers outside of the study and the reported prices charged. Workers specified whether they charged per house or per element, along with the corresponding amounts. For those charging per house, we calculated the mean cost. For those charging per element, we estimated the total by multiplying the reported cost per window or vent screened by the mean number of windows and ventilation bricks screened in our study population. We then compared these results with our study’s estimated average costs per house and per window.

## Results

### Study population characteristics

We collected resource use data for 1836 of the 2042 (88%) homes modified in the screening arm and 1822 of 1964 homes (92%) in the eave tubes arm. Socio-demographic information was obtained from cross-sectional surveys on a subset of these homes (screening: 1029; eave tubes: 1025). Households in our study area were poorer than the national average, with 39% in the wealthiest two study quintiles but only 17% in the wealthiest two national quintiles. Of 1029 homes in the screening arm, 371 (36%) were classified as “modern”, as were 389 of 1025 (38%) in the eave tubes arm (Table 1).

### Intervention costs

Amongst the 1836 homes with screening resource use data, a mean of 3.3 windows (range: 0–25) and 5.8 ventilation bricks (range: 0–20) were screened per home. Eaves were screened in < 1% of homes (n = 13) because most were already closed. The mean societal economic cost per house screened was $116 (95% CI 112–120; total cost for all homes $236,700); mean financial cost to the provider was $122 (total cost $248,227). The study covered the majority of installation costs, with household contributions accounting for < 1% for both interventions. Valuing capital costs by subtracting residual value from initial value resulted in higher financial than economic costs. Supplies and materials ($69 per house) made up 60% of societal costs, followed by labour ($34; 30%) and local transportation ($9; 8%) (Table S1).

Amongst the 1,822 homes with eave tubes resource use data, a mean of 9.2 eave tubes (range: 1–68) were installed per home. Most eave tubes were installed behind vents (93%); very few were installed directly into the wall using locally available tools (5%) or using imported specialized drills (2%). The mean societal economic cost of installation per house receiving eave tubes was 58% lower than for screening ($50; 95% CI 48–52; total cost for all homes $98,185); mean financial cost to the provider was $57 (total cost $111,688). Supplies and materials ($24 per house) made up 48% of societal costs, followed by labour ($11; 23%) and equipment ($5; 11%).

**Fig. 2:**
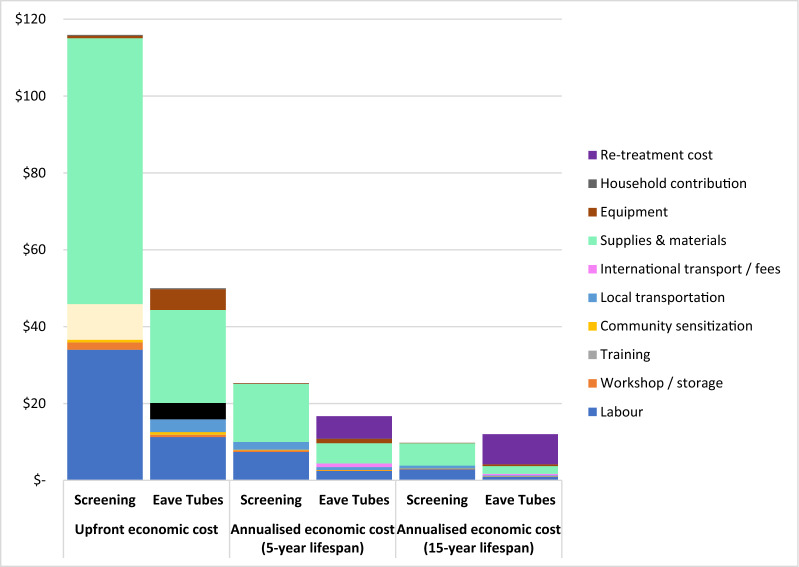
Upfront and annualized societal costs of housing modification installation and re-treatment. Annualization assumes 5.5 people per household. Installation costs estimated within the trial and annual eave tubes re-treatment costs taken from Sternberg et al*.* 2021. All costs presented in constant 2022 USD

Assuming a 5-year intervention lifespan with annual eave tubes retreatment, the annualized -cost of screening was 51% higher than eave tubes per home ($25 vs. $17) and per person ($4.59 vs. $3.03) (Table S2). Assuming a 15-year intervention lifespan with annual eave tubes retreatment, however, screening cost 19% less than eave tubes per home ($9.74 vs. $12.01) and per person ($1.77 vs. $2.19) (Fig. 2).

**Fig. 3:**
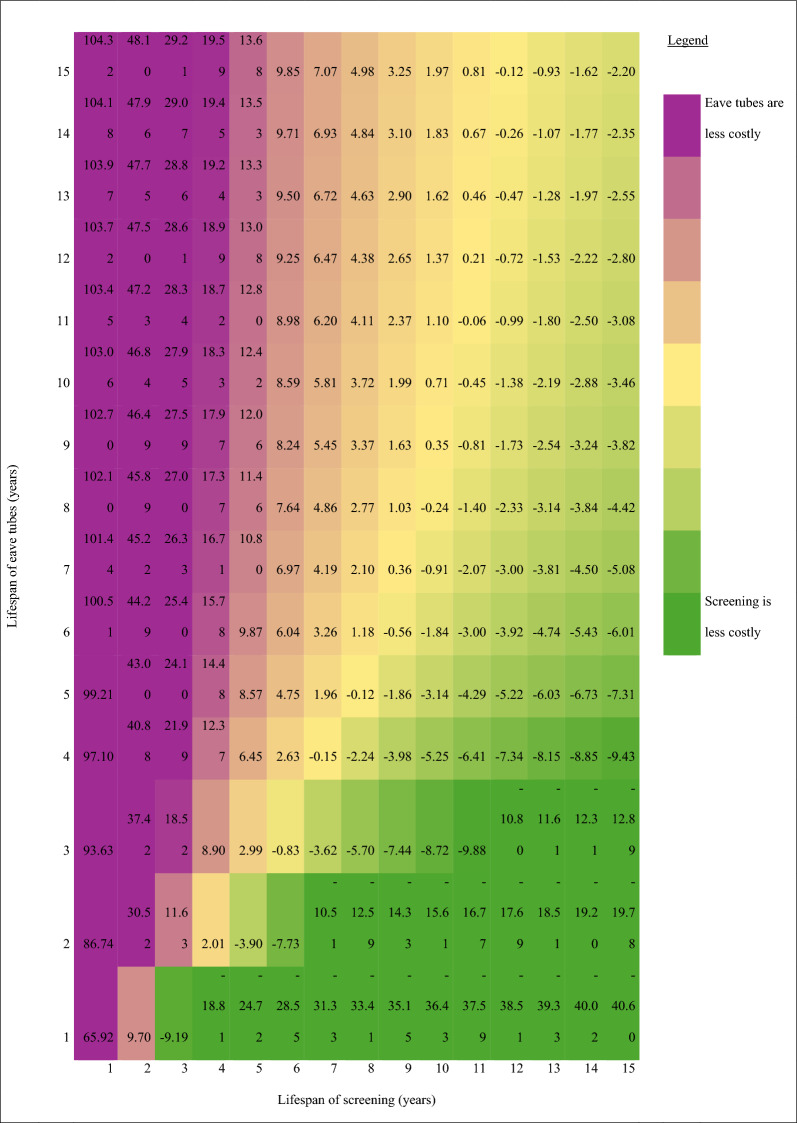
Two-way sensitivity analysis: Cost Difference between Screening and Eave Tubes over Varying Lifespans. The number in each cell represent the annualized cost of screening less the annualized cost of eave tubes, given the expected lifespan. All costs presented in constant 2022 USD

One-way sensitivity analyses showed that plausible variations in intervention lifespan, discount rate, proportion of traditional homes, daily construction worker wages, and labour requirements per house resulted in > 5% variation in the cost per person protected per year for both housing modification interventions (Table S4, Figure S7). Additionally, variations in the number of windows screened led to > 5% variation in screening costs, while changes in the number of eave tubes installed, retreatment costs, and retreatment frequency caused > 5% variation in eave tube costs. Intervention lifespan was the largest cost driver; reducing lifespan to one year increased screening costs 3.5-fold and eave tube costs two-fold. Furthermore, varying lifespan determined which intervention had higher costs over time (Fig. 3).

### Cost variation

We found that modifying modern homes was more costly than traditional homes in part because of their larger perimeters in both the screening (32 vs. 27 m) and eave tubes (32 vs. 26 m) arms, which required more screened windows (mean 4.3 vs. 2.8) and ventilation bricks (mean 7.3 vs. 5.2) and more eave tubes (mean 11.8 vs. 7.7)(Fig. S5). In the screening arm, modifying modern homes cost 49% more than traditional homes: $150 (95% CI $141–159) compared to $101 (95% CI $95–106). In the eave tubes arm, the cost was 65% higher: $66 (95% CI 61–72) for modern homes versus $40 (95% CI 38–43) for traditional homes. For eave tubes, differences in wall materials influenced installation methods: modern homes were more likely to have eave tubes installed behind ventilation bricks (94% vs. 79%) or using a drill (4% vs. 2%) and less likely to use local tools (2% vs. 20%), resulting in three times higher equipment costs ($9.56 vs. $3.04), primarily due to the use of imported specialized drilling equipment.

**Fig.4:**
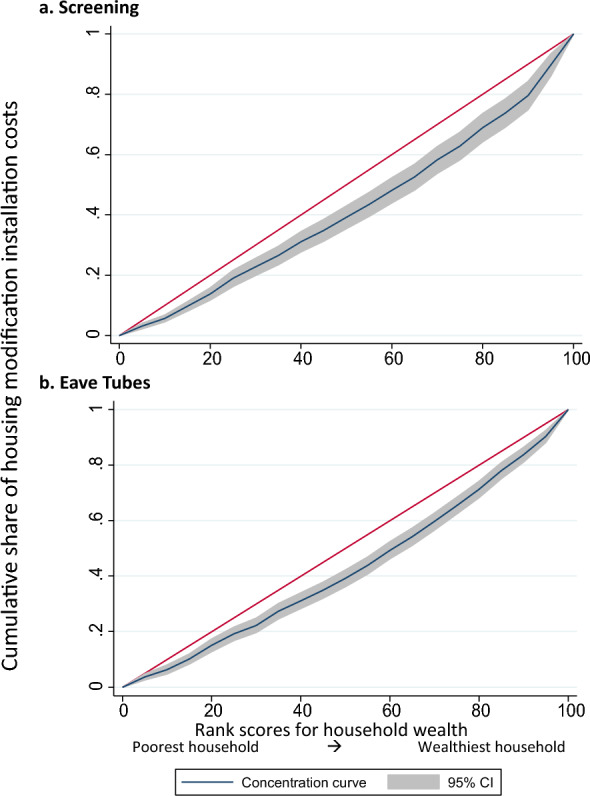
Concentration curves: Variation in housing modification costs by household wealth status. The unit of analysis is the household. The concentration indices were 0.146 for **a** screening (p < 0.0001) and 0.168 (p < 0.0001) for **b** eave tubes

Homes in the wealthiest study quintile cost 1.8 times more to modify than in the poorest quintile for both interventions (Table 2). When using national quintiles, this gap increased: screening costs were 2.2 times higher and eave tubes 3.1 times higher for homes in the wealthiest quintile compared to the poorest. Concentration indices support this finding, with concentration curves for screening (index = 0.168; p < 0.0001) and eave tubes (index = 0.146; p-value < 0.0001) significantly below the line of equality, indicating that wealthier households consumed a higher share of modification costs (Fig. 4). Although wealthier homes were more expensive to modify, the cost represented a smaller percentage of annual household consumption in the highest wealth quintiles compared to the lowest for both for screening (0.75% vs. 3.1%) and for eave tubes (0.47% vs. 1.4%).

**Table 2:** Costs of housing modification compared to annual expenditure and willingness to pay by wealth

National wealth quintile	Intervention cost^**a**^						Willingness to pay^**b**^							
	Number of observations	Installation cost		Annual household consumption	Intervention cost as % of annual consumption		Mean WTP		WTP as % of Intervention Costs		Gap between Cost and WTP		% of household respondents willing to pay cost of intervention	
		Screening	Eave tubes		Screening	Eave Tubes	Screening	Eave Tubes	Screening	Eave Tubes	Screening	Eave Tubes	Screening	Eave Tubes
All homes	2054	$118	$50	$5,943	1.99%	0.84%	$8.60	$8.16	7%	16%	$110	$42	1.52%	3.96%
Poorest	326	$69	$31	$2,202	3.12%	1.40%	$4.29	$4.25	7%	14%	$64	$27	0%	2.33%
Poor	582	$105	$41	$4,143	2.54%	0.99%	$6.27	$6.35	6%	15%	$99	$35	2.04%	2.59%
Middle	762	$132	$55	$6,208	2.12%	0.88%	$8.64	$8.53	7%	15%	$123	$47	1.05%	4.02%
Wealthy	301	$152	$61	$8,854	1.71%	0.69%	$15.00	$12.27	9%	18%	$137	$50	2.14%	8.16%
Wealthiest	83	$151	$95	$20,281	0.75%	0.47%	$20.76	$17.23	12%	18%	$130	$78	3.23%	2.08%

To understand the relative financial impact on households, we compared estimates for the cost per house modified with estimates of consumption expenditure by national quintile

All costs presented in constant 2022 USD

^**a**^We modified a total of 2084 screening homes and 1986 eave tubes homes. A subset of these homes had both demographic and resource use data collected (screening = 1029; eave tubes = 1025 homes). We used EquityTool to assign the households in our study to Uganda-wide national wealth quintiles and their associated mean per capita consumption expenditure. Consumption estimates were obtained from the World Bank PIP data set which provides mean daily consumption per person. We multiplied per capita daily consumption by the observed number of household members in those households and days in a year

^**b**^Willingness-to-pay (WTP) data obtained from the combined dataset across five timepoints and the demographic/resource use dataset, resulting in a dataset of 985 homes that received screening and 987 homes that received eave tubes. WTP values are mean values, zeros included

In univariate regression analyses for both interventions, the cost per house modified was significantly higher in homes in higher wealth quintiles or with an older head of household, more members, larger perimeter, or modern construction. In the screening intervention, costs were also significantly higher for households with a more educated and/or female head (Table S3).

In multivariable analyses for both interventions, all variables remained in the models except sex of the household head, which did not improve model fit (Table 3). In the screening model, controlling for all variables, households headed by an older individual (age > 50 years) had costs 40% higher than those with a younger head (p < 0.001). Homes in the wealthiest national quintile cost 63% more to modify than those in the poorest quintile (p < 0.001). Each additional household member increased costs by 4% (p < 0.001), and modern homes cost 9% more to modify than traditional homes (p < 0.001). In the eave tubes model, similar trends were observed for head of household age (coef.: 1.45, p < 0.001), household wealth (coef.: 2.28, p < 0.001), number of household members (coef.: 1.02, p < 0.001), and house type (coef.: 1.16, p < 0.001). Additionally, homes with a head educated beyond secondary school had 30% lower costs than those with less education (p = 0.024), a finding that contrasts with the direction observed in the univariate analyses.

**Table 3:** Multivariable analysis of factors associated with cost per house modified

			Screening (n = 920)^**b**^			Eave Tubes (n = 903)^**b**^			Mean raw costs^a^	
			Coef.^a^	p-value	95% CI	Coef.^a^	p-value	95% CI	Screening	Eave Tubes
Head of Household characteristics	Age (years)	< 30	Reference			Reference			$81.63	$34.41
		31–40	6.60	0.407	(− 8.3 to 23.8)	16.5	0.022	(2.22–32.8)	$106.12	$46.79
		41–50	21.7	0.014	(3.97–42.6)	41.6	< 0.001	(23.2–62.7)	$131.93	$60.91
		51 +	40.4	< 0.001	(21.6–62.2)	45.4	< 0.001	(28.1–65.0)	$146.44	$63.13
	Education level	Secondary or lower	Reference			Reference			$118.85	$52.01
		Higher education	23.8	0.152	(0.92–1.66)	0.70	0.024	(0.52–0.95)	$171.18	$55.51
Household characteristics	National wealth quintiles	Poorest	Reference			Reference			$68.15	$33.50
		Poor	28.6	< 0.001	(9.96–50.5)	14.4	0.048	(0.10–30.7)	$103.94	$41.67
		Middle	60.6	< 0.001	(36.4–89.2)	39.5	< 0.001	(20.9–60.8)	$134.64	$59.34
		Wealthy	82.7	0.003	(47.6–126.)	35.5	0.001	(13.0–62.4)	$169.77	$59.91
		Wealthiest	63.8	0.001	(17.7–128.)	128	< 0.001	(77.3–194.)	$142.52	$101.85
	Number of household members	continuous	4.08	< 0.001	(1.71–6.60)	2.42	0.024	(0.30–4.60)	$120.39	$52.12
	House Perimeter (meters)	continuous	1.20	0.000	(0.90–1.51)	0.80	< 0.001	(0.50–1.10)	$120.39	$52.12
	House type	Traditional	Reference			Reference			$100.50	$41.99
		Modern	9.96	0.148	(− 3.3 to 25.2)	16.9	0.010	(3.87–31.7)	$154.80	$68.21

^a^Costs were log-transformed to address model heteroskedasticity. The coefficients from the regression model are interpreted as percentage changes in the dependent variable

^**b**^Only homes that had data on costs, demographic information and household size (perimeter) were included

^c^To translate the log-transformed coefficients back into raw cost values, we applied a smearing estimator[48]

### Willingness to pay

At all three time points, at least 75% of respondents were willing to pay something for each intervention, with 74–79% ranking screening as their most preferred option (Table S5). Including those who were unwilling to pay, the mean WTP for screening was $8.69 pre-installation, $8.79 at 12-month follow-up, and $5.79 at 18-month follow-up. For eave tubes, the mean WTP was $7.62 pre-installation, $7.67 at 12 months, and $4.75 at 18 months. WTP remained similar at pre-installation and 12 months post-installation but decreased significantly at 18 months. At 18 months, respondents were willing to pay $2.34 (27%) less for screening (p < 0.001) and $2.44 (32%) less for eave tubes (p < 0.001) compared to pre-installation. This declining trend was consistent across both interventions in the eave tubes and control arms. However, respondents in the screening arm showed an increase in WTP for screening over time, rising from $6.66 at baseline to $6.91 at 18 months.

In the multivariable analyses (Table S6-S8) across six two-part models (three time points for each of the two interventions), all variables remained in the models except sex of the household head, which did not improve model fit. In all six models, household wealth and the number of residents significantly drove variation in the overall WTP marginal effects (combining both parts of the model), with higher wealth quintiles and larger households having higher WTP. For example, at 18 months post-installation and assessing WTP for screening, respondents from households in the highest wealth quintile were willing to pay $8.33 more than those in the lowest (p < 0.001) and an additional household member increased WTP by $0.47 (p = 0.001).

The price elasticity of demand for screening ranged from − 0.36 to − 0.48, and for eave tubes from − 0.34 to − 0.46, indicating inelastic demand for housing modifications (Table S9). Assuming constant elasticity, a 10% increase in modification costs resulted in only a 3–5% decrease in quantity demanded, showing that respondents were relatively unresponsive to price changes. The large gap between modification costs and WTP suggests that the product is perceived as overpriced, with an $8 WTP suggesting low demand at current prices. Demand for both interventions became more elastics over time, with slightly higher price sensitivity observed in wealthier households compared to poorer ones.

### Financing gap

**Fig. 5:**
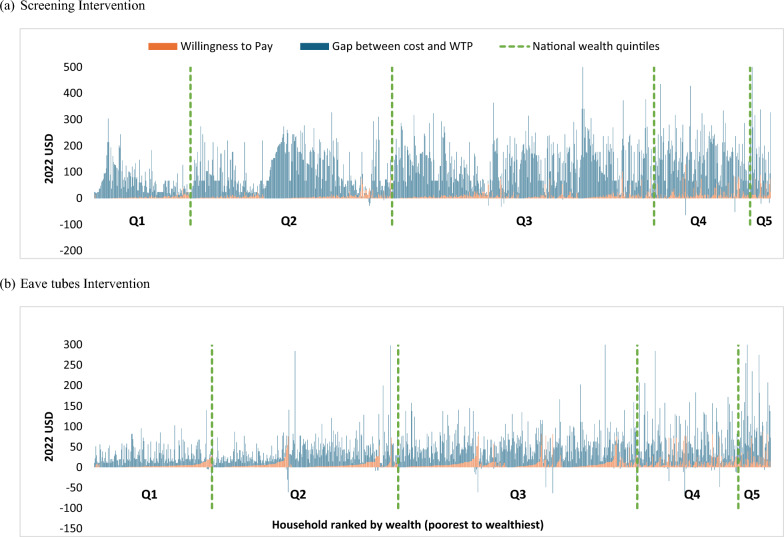
Financing gap between willingness to pay and cost of housing modification by household wealth. **a** Screening Intervention. **b** Eave tubes Intervention. Each vertical bar represents a household. Households are ranked from poorest (left) to richest (right), with dotted lines delineating national wealth quintiles. Each household’s WTP (orange) and gap between cost and WTP (dark blue) are shown. Households willing to pay more than it cost to modify their house are shown with negative values for the financing gap

Few households were willing to cover the full cost of either intervention (Fig. 5). For screening, only 15 of 987 (1.5%) households were willing to pay the full cost of house modifications, ranging from 3.2% (3/31) in the wealthiest quintile to none (0/140) in the poorest (Table 3). On average, households were willing to pay only 13.2% of the total screening costs, with a mean gap of $110 between WTP and actual costs (range: − $64 to $643). For eave tubes, 4.0% (39/985) of households were willing to pay the full cost, including 2.9% (1/48) in the wealthiest quintile, 2.3% (4/172) in the poorest, and 8.16% (12/147) in the second wealthiest quintile. On average, households were willing to pay 24.3% of the modification costs, with a mean gap of $42 between WTP and costs (range: − $63 to $430).

### Organic uptake outside study

Nine months after installation of housing modifications in the study, 37% of workers (33/116) reported installing screening in a total of 166 homes outside the study, charging an average of $123 per house (range: $6–$1211) (Table S10). Mean charges were $23 per window screen and $5 per vent screen, leading to an estimated average cost of $104 per home based on study-observed window and vent counts. Household size and wealth data were not collected, and eave tubes were not installed because they were not available in the local market.

## Discussion

We found that the mean societal economic cost per house screened ($116) was more than twice the cost of eave tube installation ($50), and that wealthier homes were significantly more expensive to modify because of their size and construction materials. Although upfront costs were relatively high, the mean annualized costs per person protected (screening: $4.22; eave tubes: $3.03) were comparable to other malaria control measures, such as LLINs ($1.39, range: $1.09–$1.83, USD 2022), IRS ($5.70, range: $2.75–$15.93), and chemoprevention (range: $0.61–$6.89) [54]. Most households (> 70%) had a positive WTP for the interventions but covered only 7–16% of costs, with WTP declining over time except for screening in the screening arm. Wealthier households were willing to contribute a higher share of costs, but few, mainly the wealthiest, were willing to pay the full price (0.5–3% of annual consumption), highlighting a substantial financing gap. Finally, the low uptake of housing modifications outside the study, where costs were high ($123 per home), raises uncertainty about whether costs differed inside versus outside the study area.

A major strength of our research was the collection of resource use data from 94% of the 4000 modified homes, allowing for a detailed analysis of cost variation, which is often absent in other studies. Our study is the first to quantify WTP for screening and the only to assess WTP for eave tubes. While WTP studies have become less common in malaria research due to the widespread provision of free interventions, many policymakers view housing modification as requiring household investment. Our use of the ‘structured haggling’ methodology, consistent with best practices for sub-Saharan Africa [39, 55], offers the advantage of yielding higher response rates compared to open-ended questions (e.g. “What are you willing to pay for X?”). By collecting WTP and cost data from the same households, we were able to assess financing gaps, an area not previously explored.

Our study has limitations. With only one year of follow-up, we could not assess eave tubes re-treatment or replacement costs or intervention lifespan. Sensitivity analyses showed that lifespan assumptions affected which intervention cost more over time and whether the cost per person protected per year is comparable to other malaria control strategies. Owing to time and resource constraints, we estimated household consumption by combining an asset-based wealth index, the EquityTool, and consumption percentiles [29]. While this approach introduces uncertainty, potentially leading to under- or over-estimates of the intervention’s cost as a percentage of annual consumption, it does not affect the equity assessments across study populations (e.g., concentration indices and curves). Valuing productivity losses using mean monthly rural household consumption ($148 per month) may not accurately reflect the opportunity cost of time for our study population; however, there is no consensus on a gold standard [56] and alternatives such as the official minimum wage ($1.60 per month)[57], GDP per capita ($80 per month)[27], and the local market rate for unskilled labour (potentially $108 per month) also have limitations. For equity analyses, we could only use the 60% of our cost dataset for which survey data on socio-demographic characteristics were available; however, this rich sub-set comprising 1832 households is representative of households in Uganda with children < 15 years, who are the most vulnerable to malaria.

Our mean cost estimates for both interventions are within the range reported in the literature, though variations in home types, intervention scope, and cost components make this range broad. A Côte d’Ivoire study found that the combined implementation of eave tubes and screening cost $240 USD (2018) per home, including 6 rounds of retreatment, with $96 for screening installation and $95 for eave tube installation [22]. The authors anticipated lower costs for separate interventions, which our study confirms for eave tubes ($50 per installation). While no other studies have estimated eave tube costs, five have reported screening costs. Those accounting only for labour and materials reported lower costs ($9 per homes [19],) as did studies without detailed cost scope ($34 per home [16],). Studies with comprehensive cost capture aligned more closely with our findings, despite differences in intervention scope ($42 per home, [15]; $94 per home, [17]; $77 per home, [18]). Our mean WTP estimates were higher than previously reported for other malaria interventions, even when adjusted to 2022 USD [58].

Trial-based analyses may over- or underestimate costs, but the similarity between our estimated screening cost per home ($116) and the prices reported by local construction workers ($123) supports the reliability of our findings. However, households who paid to screen their homes were likely wealthier, and market prices may not fully reflect costs, as the market is not perfectly competitive. Real-world implementation could lower costs through economies of scale or other efficiencies could reduce actual costs, increasing affordability. Labour, supplies, and materials accounted for 89% of screening costs, a proportion likely reflective of actual conditions, since all materials were locally sourced and construction worker wages aligned with prevailing market rates.

To support the application of our findings across diverse settings with different housing construction practices, we provided cost breakdowns by housing type and installation method. For instance, 38% of homes in our study were classified as “modern”, lower than the 50% reported for Uganda and sub-Saharan Africa in 2015 [59], allowing for adjustments in future models. Our study focused on retrofitting rather than integrating modifications during initial construction or implementing screening piecemeal over time, which may have different cost implications. Finally, the modification design and techniques in our trial were costly, but simpler alternatives might be feasible, such as non-openable screens, could reduce labour and material costs while simplifying installation, though they may be less appealing.

Our findings surface key considerations for for equity in scale-up. While wealthier homes cost more to modify, the costs represent a smaller proportion of household consumption. Without targeted subsidies or financing strategies, implementation may exacerbate inequities. Future scale-up strategies should consider targeting poorer households or housing types linked to higher malaria risk. Demand-side interventions to increase WTP and awareness may also be critical, given observed price inelasticity and limited market uptake.

Future research will examine the cost-effectiveness and equity impacts of screening and eave tubes as malaria control interventions, enabling comparisons with other public health strategies. While our focus was on malaria prevention, housing modifications provide additional health and social benefits that warrant further exploration. Multisectoral interventions remain promising, but funding constraints require further research on implementation challenges, particularly assessing real-world costs and scaling pathways.

## Conclusions

Housing modifications such as screening and eave tubes have relatively high upfront costs but annual costs per person protected that are comparable to other malaria control strategies. However, households, particularly the poorest, are largely unwilling or unable to pay the full cost of these modifications. Our findings highlight a significant financing gap and limited organic market uptake, suggesting that equitable and sustainable scale-up will require public or donor financing and/or innovative approaches such as targeted subsidies, co-payment models, or micro-financing. Given the inelastic demand observed, supply-side subsidies alone are unlikely to ensure equitable access. Demand-generation efforts and multisectoral partnerships will be critical to scale up these interventions. These findings offer timely evidence for policymakers and funders exploring integrated, equity-focused malaria control strategies in Uganda and other high-burden settings.

## Supplementary Information

Supplementary Material 1.

## Data Availability

The data underlying this article will be shared on reasonable request to the corresponding author.

## Abbreviations

CI: Confidence interval
GDP: Gross domestic product
IRS: Indoor residual spraying
LLIN: Long-lasting insecticidal net
PBO: Piperonyl butoxide
UGX: Ugandan shillings
USD: United States Dollar
WTP: Willingness to pay
WHO: World Health Organization
