# Supplementary material for: Housing modification to prevent malaria in Uganda: an analysis of costs, willingness to pay, and equity
Source: Malar J. 2025 Dec 24;25:60. doi: 10.1186/s12936-025-05757-0 (PMC12837981; doi:10.1186/s12936-025-05757-0)
Supplement: Supplementary file 1 — Supplementary Material 1. [file 12936_2025_5757_MOESM1_ESM.docx]

Supplementary Materials

[1. Methods 2](#_Toc188398616)

[Supplementary Figure S1 Timeline of study activities 2](#_Toc188398617)

[Supplementary Figure S2 Map of study area 3](#_Toc188398618)

[Supplementary Figure S3 Data cascade for construction worker survey 4](#_Toc188398619)

[Supplementary Figure S4: Schematic and flow of willingness to pay questions 5](#_Toc188398620)

[2. Cost Results 6](#_Toc188398621)

[2.1. Costs 6](#_Toc188398622)

[Supplementary Table S1 Installation costs of housing modification 6](#_Toc188398623)

[Supplementary Table S2 Annualized societal costs of housing modification installation and re-treatment 7](#_Toc188398624)

[Supplementary Figure S5 Mean costs per house modified by cost category, intervention, and housing type 8](#_Toc188398625)

[Supplementary Figure S6 Variation in intervention installation costs by household wealth status 9](#_Toc188398626)

[2.2. Regression analysis 10](#_Toc188398627)

[Supplementary Table S3 Univariate regression analysis of cost per house modified 10](#_Toc188398628)

[2.3. Sensitivity analysis 11](#_Toc188398629)

[Supplementary Table S4: Sensitivity analysis of annualized societal cost per home modified input parameters, cost outputs and justification 11](#_Toc188398630)

[Supplementary Figure S7: Tornado diagram of deterministic sensitivity analysis for annualized societal cost per home modified for (a) screening and (b) eave tubes 14](#_Toc188398631)

[3. Willingness-to-Pay Results 16](#_Toc188398632)

[3.1. WTP estimates 16](#_Toc188398633)

[Supplementary Table S5 Household mean willingness to pay for housing modification installation and re-treatment 16](#_Toc188398634)

[3.2 Regression analyses 17](#_Toc188398635)

[Supplementary Table S6 Willingness to pay one-way regression results 17](#_Toc188398636)

[Supplementary Table S7 Two-part model for Screening WTP at three timepoints 18](#_Toc188398637)

[Supplementary Table S8 Two-part model for Eave Tubes WTP at three timepoints 21](#_Toc188398638)

[3.2. Elasticities 24](#_Toc188398639)

[Supplementary Table S9 Point elasticities for housing modifications 24](#_Toc188398640)

[4. Organic uptake outside study 25](#_Toc188398641)

[Supplementary Table S10 Construction Worker Survey Results 25](#_Toc188398642)

# Methods

### Supplementary Figure S1 Timeline of study activities

*CSS = Cross-sectional survey*

|  | 2020 | 2021 | | | | | | | | | | | | 2022 | | | | | | | | | | | | 2023 | | | | | |
| --- | --- | --- | --- | --- | --- | --- | --- | --- | --- | --- | --- | --- | --- | --- | --- | --- | --- | --- | --- | --- | --- | --- | --- | --- | --- | --- | --- | --- | --- | --- | --- |
|  | Dec | Jan | Feb | Mar | Apr | May | Jun | Jul | Aug | Sept | Oct | Nov | Dec | Jan | Feb | Mar | Apr | May | Jun | Jul | Aug | Sept | Oct | Nov | Dec | Jan | Feb | Mar | Apr | May | Jun |
| **Construction Activities** | Phase I Screening Installation |  |  |  |  |  |  |  |  |  |  |  | Phase II Screening Installation | | | | |  |  |  |  |  |  |  |  |  |  |  |  |  |  |
|  |  |  |  |  |  |  |  |  |  |  |  |  |  |  |  |  |  |  |  |  |  |  |  |  |  |  |  |  |  |  |  |
|  | Phase I Eave Tubes Installation |  |  |  |  |  |  |  |  |  |  |  |  |  |  | Phase II Eave Tubes Installation | |  |  |  |  |  |  |  |  |  |  |  |  |  |  |
|  |  |  |  |  |  |  |  |  |  |  |  |  |  |  |  |  |  |  |  |  |  |  |  |  |  |  |  |  |  |  |  |
| **Data Collection** | Phase I Baseline  CSS |  |  |  | Phase I Endline CSS |  |  |  |  |  | Phase II Baseline CSS | |  | Cohort Baseline Survey | | | |  |  |  |  |  | Phase II 12-month CSS | |  |  |  | Phase II Endline CSS | | Cohort Endline Survey | |
|  |  |  |  |  |  |  |  |  |  |  |  | |  |  | | | |  |  |  |  |  |  | |  |  |  |  | |  | |
|  |  |  |  |  |  |  |  |  |  |  |  | |  |  | | | |  |  |  |  |  |  | Construction Worker  Survey |  |  |  |  | |  | |

### Supplementary Figure S2 Map of study area


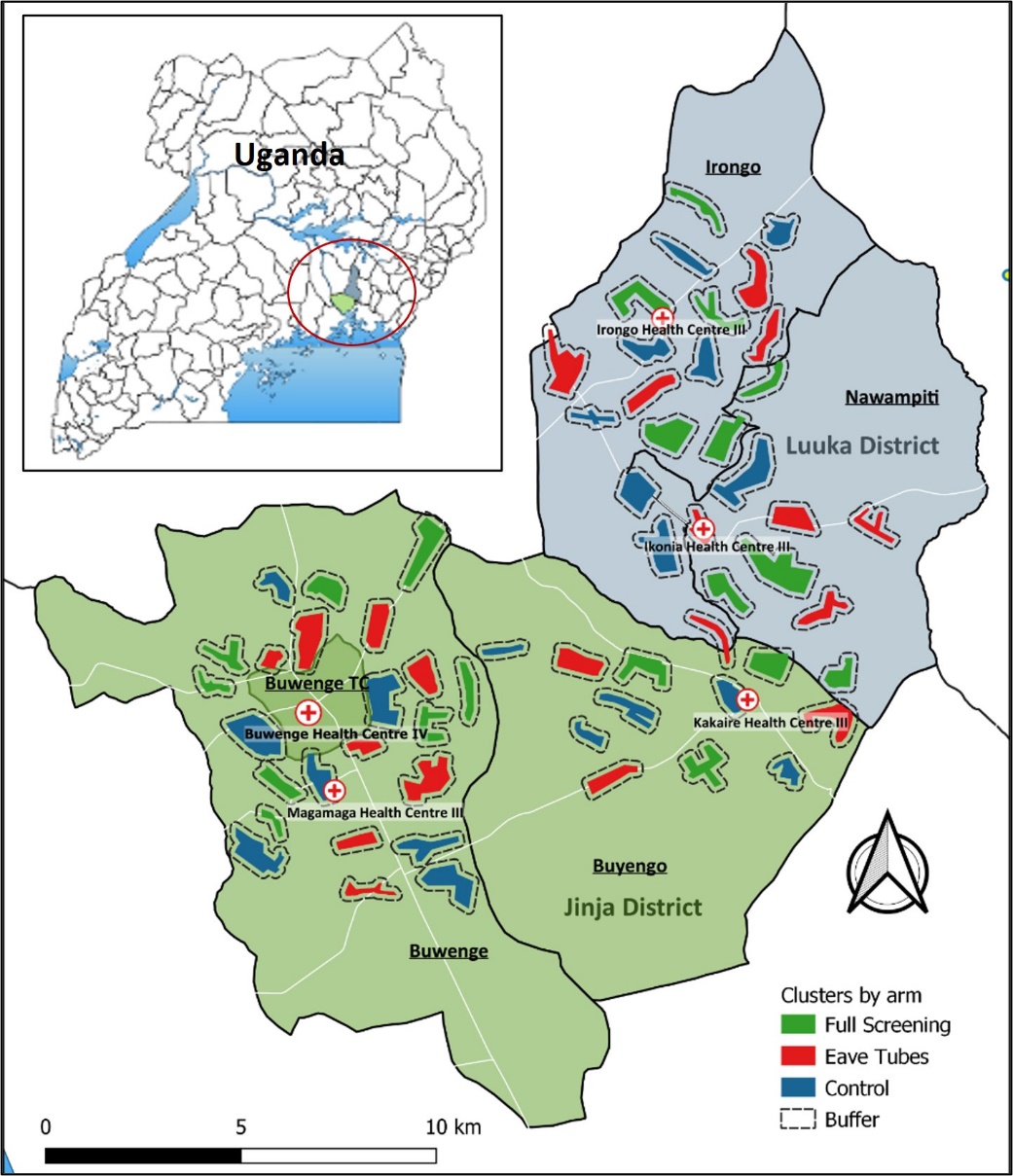


### Supplementary Figure S3 Data cascade for construction worker survey

### Supplementary Figure S4: Schematic and flow of willingness to pay questions

*Schematic populated with values for screening intervention. Exchange rate: 1 USD = 3961 UGX.*

| **Question 1:** Are you willing to pay 280,000 UGX for screening?  **Question 2**: What is the maximum amount you are willing to pay?  **Question 3**: What if the price is 210,000 UGX, would you be willing to pay?  **Question 4**: What is the price is 140,000 UGX, would you be willing to pay?  **Question 5**: What really is the maximum amount you are willing to pay for screening?  **Question 6:** The amount that you have quoted is too low and cannot cover the cost of the screening, and so you will have to increase the amount if you really want to buy the screening. So, what is the final maximum amount you are willing to pay for screening?    **Question 7:** If, due to inflation or other uncertainties, the price of the screening increases, what is the maximum amount you are very certain to pay? |  |
| --- | --- |

# Cost Results

## Costs

### Supplementary Table S1 Installation costs of housing modification

*All costs present in constant 2022 USD*

| **Economic Costs** | |  |  |  |
| --- | --- | --- | --- | --- |
| **Cost Category** | **Screening** | | **Eave Tubes** | |
|  | **Total** | **Per house USD** | **Total** | **Per House USD** |
| Labour | 69,467.25 | 34.02 | 22,102.51 | 11.25 |
| Workshop / Storage | 3,730.88 | 1.83 | 1,080.70 | 0.55 |
| Training | 121.92 | 0.06 | 175.97 | 0.09 |
| Community Sensitization | 1,413.28 | 0.69 | 1,342.17 | 0.68 |
| Local Transportation | 18,946.79 | 9.28 | 6,492.59 | 3.31 |
| International transport/fees | 0 | 0 | 8,375.18 | 4.26 |
| Supplies & materials | 141,296.99 | 69.20 | 47,535.96 | 24.20 |
| Equipment | 1,372.71 | 0.67 | 10,619.88 | 5.41 |
| **Provider Costs** | **236,349.82** | **115.74** | **97,724.96** | **49.76** |
| **Household Costs** | **349.92** | **0.17** | **460.06** | **0.23** |
| **Societal Costs** | **236,699.74** | **115.92** | **98,185.02** | **49.99** |
| **Financial Costs** | | |  |  |
| **Cost Category** | **Screening** | | **Eave Tubes** | |
|  | **Total** | **Per house USD** | **Total** | **Per House USD** |
| Labor | 69,467.25 | 34.02 | 22,102.51 | 11.25 |
| Workshop / Storage | 3,882.60 | 1.90 | 1,080.70 | 0.55 |
| Training | 121.92 | 0.06 | 175.97 | 0.09 |
| Community Sensitization | 1,413.28 | 0.69 | 1,342.17 | 0.68 |
| Local Transportation | 29,661.49 | 114.53 | 11,534.80 | 5.87 |
| International transport/fees | 0 | 0 | 8,375.18 | 4.26 |
| Supplies & materials | 141,296.99 | 69.20 | 47,535.96 | 24.20 |
| Equipment | 2,383.62 | 1.17 | 19,540.84 | 9.95 |
| **Provider Costs** | **248,227.14** | **121.56** | **111,5688.13** | **56.87** |

### Supplementary Table S2 Annualized societal costs of housing modification installation and re-treatment

*Analysis assumes 5.5 people per household and a 3% discount rate. Installation costs taken from the trial and eave tubes re-treatment costs taken from Sternberg et al. 2021. All costs present in constant 2022 USD.*

|  | **Screening** | | **Eave Tubes** | |
| --- | --- | --- | --- | --- |
|  | **Financial cost** | **Annualized economic cost** | **Financial Cost** | **Annualized economic cost** |
| **Assuming 5-year lifespan** |  |  |  |  |
| Installation cost | 115.92 |  | 49.99 |  |
| Retreatment costs |  |  | 26.60 |  |
| Total costs | 115.92 |  | 76.59 |  |
| Annual cost of protection | 23.18 | 25.27 | 15.32 | 16.69 |
| Annual cost per person protected | 4.22 | 4.59 | 2.79 | 3.03 |
| **Assuming 15-year lifespan** |  |  |  |  |
| Installation cost | 115.92 |  | 49.99 |  |
| Retreatment costs |  |  | 93.10 |  |
| Total costs | 115.92 |  | 143.09 |  |
| Annual cost of protection | 7.73 | 9.74 | 9.54 | 12.02 |
| Annual cost per person protected | 1.41 | 1.77 | 1.73 | 2.19 |

### Supplementary Figure S5 Mean costs per house modified by cost category, intervention, and housing type

*Economic costs of installation using a societal perspective are presented in 2022 USD. “Other” includes training, community sensitization, workshop and storage costs. The “all” homes mean costs were calculated using top-down methods, 2,084 screening homes and 1,986 eave tubes homes. The modern and traditional mean costs were calculated using micro-costing methods on homes with survey data available (screening: 1029 homes; eave tubes: 1025 homes).*

### Supplementary Figure S6 Variation in intervention installation costs by household wealth status

*Variation in the economic costs of installation (societal perspective) by with-in study wealth quintiles and National wealth quintiles. We modified a total of 2,084 screening homes and 1,986 eave tubes homes. A subset of these homes had both demographic and micro-costing data collected (screening=1,029; eave tubes=1,025 homes). We constructed study wealth quintiles using principal components analysis. We used EquityTool to assign the households in our study to Uganda-wide national wealth, All costs presented in in 2022 USD.*

## Regression analysis

### Supplementary Table S3 Univariate regression analysis of cost per house modified

*The economic costs of installation using a societal perspective is the dependent variable. All costs presented in in 2022 USD.*

|  |  |  | **Screening n=1,029*** | | | **Eave Tube n=1,025*** | | |
| --- | --- | --- | --- | --- | --- | --- | --- | --- |
|  |  |  | Coef. | p-value | 95% CI | Coef. | p-value | 95% CI |
| **Head of Household characteristics** | **Age** | **<30 years** | *reference* | | | *reference* | | |
|  |  | **0-40** | 21.21 | 0.004 | (6.79 - 35.64) | 10.07 | 0.007 | (2.81 - 17.32) |
|  |  | **41-50** | 54.81 | <0.001 | (40.1 - 69.54) | 23.8 | <0.001 | (16.32 - 31.29) |
|  |  | **51+** | 62.27 | <0.001 | (48.71 - 75.83) | 25.49 | <0.001 | (18.61 - 32.38) |
|  | **Education Level** | **Secondary or lower** | *reference* | | | *reference* | | |
|  |  | **Higher education** | 32.36 | 0.027 | (3.66 - 61.06) | 6.9 | 0.433 | (-10.36 - 24.17) |
|  | **Gender** | **Female** | *reference* | | | *reference* | | |
|  |  | **Male** | -15.18 | 0.008 | (-26.44 - -3.91) | -5.63 | 0.062 | (-11.55 - 0.27) |
| **Household characteristics** | **National Wealth Quintiles** | **Poorest** | *reference* | | | *reference* | | |
|  |  | **Poor** | 36.74 | <0.001 | (21.48 - 52) | 10.02 | 0.008 | (2.66 - 17.38) |
|  |  | **Middle** | 62.85 | <0.001 | (48.23 - 77.47) | 23.87 | <0.001 | (16.84 - 30.91) |
|  |  | **Wealthy** | 83.16 | <0.001 | (65.45 - 100.86) | 30.22 | <0.001 | (21.77 - 38.68) |
|  |  | **Wealthiest** | 82.59 | <0.001 | (52.95 - 112.23) | 63.87 | <0.001 | (51.67 - 76.07) |
|  | **Number of household members** | **Continuous** | 6.33 | <0.001 | (4.02 - 8.64) | 3.02 | <0.001 | (1.87 - 4.18) |
|  | **House Perimeter** | **Continuous** | 1.49 | <0.001 | (1.18 - 1.79) | 0.72 | <0.001 | (0.54 - 0.89) |
|  | **House type** | **traditional** | *reference* | | | *reference* | | |
|  |  | **modern** | 48.83 | <0.001 | (38.87 - 58.78) | 0 | <0.001 | (26.25 - <0.001) |

## Sensitivity analysis

### Supplementary Table S4: Sensitivity analysis of annualized societal cost per home modified input parameters, cost outputs and justification

1. Input parameters and justification

| **Variable Description** | **Best estimate** | **Lower estimate** | **Upper estimate** | **Annualized cost per home** | **% change** | **Annualized cost per home** | **% change** | **Justification** |
| --- | --- | --- | --- | --- | --- | --- | --- | --- |
| ***Screening Intervention*** |  |  |  |  |  |  |  |  |
| Discount rate for annualization | 3% | 1% | 5% | 23.88 | -6% | 26.777 | 6% | IDSI reference case for LMIC (1) |
| Lifespan of intervention (years) | 5 | 1 | 15 | 115.92 | 359% | 9.737 | -61% | Author’s assumption [Upper: authors’ assumption; Lower: CIV trial (2)] |
| Percentage of homes with traditional construction | 64% | 73% | 27% | 24.31 | -4% | 29.22 | 16% | Observed in study [Upper: percentage of rural homes with mud/dung floors 73%; Lower: percentage of rural homes with finished roof(3)] |
| Number of windows screened per home | 3.34 | 2 | 5 | 15.44 | -39% | 38.02 | 50% | Observed in study [Upper: quartile 1; Lower: quartile 3] |
| Value of a day lost of productivity for household costs | $2.40 | $2.17 | $6.70 | 25.26 | 0% | 25.34 | 0% | Rural median household consumption (4) [Upper: GDP per adult (5); Lower: rural median household monthly income (4)] |
| Wood cost per house | $33.63 | $29.59 | $37.67 | 24.38 | -4% | 26.15 | 3% | Observed in study [Upper and lower: price variation recorded throughout study period: +/- 12%] |
| Screws/nails cost per house | $11.13 | $9.46 | $12.80 | 24.91 | -1% | 25.63 | 1% | Observed in study [Upper and lower: price variation recorded throughout study period: +/- 15%] |
| Mesh cost per house | $7.00 | $5.60 | $8.40 | 25.18 | 0% | 25.36 | 0% | Observed in study [Upper and lower: price variation recorded throughout study period: +/- 20%] |
| Fuel cost per house | $0.99 | $0.84 | $1.14 | 25.24 | 0% | 25.3 | 0% | Mean price observed in study [Upper & Lower: Price variation reported within study period(6) |
| Private hire cost per house | $6.67 | $5.00 | $8.34 | 24.90 | -1% | 25.63 | 1% | Observed in study [Upper and lower: price variation recorded throughout study period: +/- 25%] |
| Daily wage rate for construction workers | $7.62 | $5.72 | $9.53 | 23.55 | -7% | 26.99 | 7% | Wage rate proportional to the days worked for three types of construction workers employed by the study [Upper and lower estimates based on informal discussion with workers: +/-25%] |
| Amount of labour per house (days) | 3.87 | 1.94 | 5.81 | 21.83 | -14% | 28.71 | 14% | Observed in study [Upper and lower: +/50% to represent the high level of uncertainty because this was a trial setting] |
| ***Eave Tubes Intervention*** |  |  |  |  |  |  |  |  |
| Discount rate for annualization | 3% | 1% | 5% | 15.77 | -6% | 17.69 | 6% | IDSI reference case for LMIC (1) |
| Lifespan of intervention (year) | 5 | 1 | 15 | 49.99 | 200% | 12.02 | -28% | Author’s assumption [Upper: authors’ assumption; Lower: CIV trial (2)] |
| Percentage of homes with traditional construction | 62% | 73% | 27% | 16.07 | -4% | 18.68 | 12% | Observed in study [Upper: percentage of rural homes with mud/dung floors 73%; Lower: percentage of rural homes with finished roof(3)] |
| Number of eave tubes installed | 9.18 | 4 | 12 | 12.63 | -24% | 19.4 | 16% | Observed in study [Upper: quartile 1; Lower: quartile 3] |
| Value of a day lost of productivity for household costs | $2.40 | $2.17 | $6.70 | 16.69 | 0% | 16.77 | 0% | Rural median household consumption (4) [Upper: GDP per adult (5); Lower: rural median household monthly income (4)] |
| PVC pipe cost per house | 4.85 | 4.12 | 5.58 | 16.53 | -1% | 16.86 | 1% | Observed in study [Upper and lower: Due to the COVID-19 pandemic, our study experienced supply chain issues which may have affected the price of imported materials such as PVC pipes. We accounted for this by adjusting observed study prices +/- 15%] |
| Cost of inserts per house | 13.52 | 10.14 | NA | 15.96 | -4% | NA | NA | We assume that if there is a wide uptake of eave tubes, there will be a price reduction for the inserts due to economies of scale [Lower estimate: -25%] |
| Screws/nails cost per house | 0.49 | 0.42 | 0.56 | 16.68 | 0% | 16.71 | 0% | Observed in study [Upper and lower: price variation recorded throughout study period: +/- 15%] |
| Fuel cost per house | 1.8 | 1.53 | 2.07 | 16.64 | 0% | 16.76 | 0% | Mean price observed in study [Upper & Lower: Price variation reported within study period(6) |
| Private hire cost per house | 2.04 | 1.53 | 2.55 | 16.58 | -1% | 16.81 | 1% | Observed in study [Upper and lower: price variation recorded throughout study period: +/- 25%] |
| Daily wage rate for construction workers | $7.62 | $5.72 | $9.53 | 16.19 | -3% | 17.20 | 3% | Wage rate proportional to the days worked for three types of construction workers employed by the study [Upper and lower estimates based on informal discussion with workers: +/-25%] |
| Amount of labour per house (days) | 0.90 | 0.45 | 1.35 | 15.47 | -7% | 17.92 | 7% | Observed in study [Upper and lower: +/50% to represent the high level of uncertainty because this was a trial setting] |
| Eave Tubes annual retreatment costs (cost per round) | 6.65 | 4.655 | NA | 14.96 | -10% | NA | NA | Point and lower estimate (30% reduction) assumption from *Sternberg et al.* |
| Frequency of eave tube retreatment (month) | 12.00 | NA | 18.00 | NA | NA | 13.79 | 17% | Current In2Care guidance [Upper: In2Care report on potential longer half-life of insecticide(7)] |

1. Wilkinson T, Sculpher MJ, Claxton K, Revill P, Briggs A, Cairns JA, et al. The International Decision Support Initiative Reference Case for Economic Evaluation: An Aid to Thought. Value in Health [Internet]. 2016;19(8):921–8. Available from: http://dx.doi.org/10.1016/j.jval.2016.04.015

2. Sternberg ED, Cook J, Alou LPA, Assi SB, Koffi AA, Doudou DT, et al. Impact and cost-effectiveness of a lethal house lure against malaria transmission in central Côte d’Ivoire: a two-arm, cluster-randomised controlled trial. The Lancet. 2021 Feb 27;397(10276):805–15.

3. Ugandan Ministry of Health, National Malaria Control Division, Uganda Bureau of Statistics. Uganda Malaria Indicator Survey 2018-19 [Internet]. Kampala, Uganda and Rockville, Maryland, USA; 2020 Mar [cited 2021 Sep 7]. Available from: https://dhsprogram.com/publications/publication-mis34-mis-final-reports.cfm

4. Ugandan Bureau of Statistics. Uganda National Survey Report 2019-2020 [Internet]. 2021. Available from: https://www.ubos.org/wp-content/uploads/publications/09_2021Uganda-National-Survey-Report-2019-2020.pdf

5. World Bank. GDP (current US$) - Uganda [Internet]. 2023. Available from: https://data.worldbank.org/indicator/NY.GDP.MKTP.CD?locations=UG

6. Uganda Bureau of Statistics. National Quarterly Average Fuel Prices [Internet]. 2022 [cited 2025 Jan 20]. Available from: https://www.ubos.org/wp-content/uploads/statistics/Average_Retail_Prices_per_litre_of_selected_Petroleum_Products_(Ugx)_E6_(2).xls

7. In2Care. Efficacy Report 12 Month Efficacy Deltamethrin on In2Care Eave Tube Disks [Internet]. 2018 [cited 2023 May 9]. Available from: www.in2care.org/marketing/EaveTubes/Efficacy_Report_12_Month_Efficay_Deltamethrin_on_In2Care_EaveTube_discs.pdf

###

### Supplementary Figure S7: Tornado diagram of deterministic sensitivity analysis for annualized societal cost per home modified for (a) screening and (b) eave tubes

1. **Screening**
2. **Eave tubes**

# Willingness-to-Pay Results

## WTP estimates

### Supplementary Table S5 Household mean willingness to pay for housing modification installation and re-treatment

*Mean willingness to pay for housing modification continuous monetary measure. Estimates were calculated both including and excluding responses indicating zero WTP. All costs in constant 2022 USD.*

| CROSS-SECTIONAL SURVEY ROUND | | | | 1: Pre-installation  *screening arm = 500; eave tubes arm = 500; control arm = 500* | | 2: 12-Month follow-up  *screening arm = 482; eave tubes arm = 443; control arm = 575* | | 3: 18-Month follow-up  *screening arm = 500; eave tubes arm = 500; control arm = 500* | |
| --- | --- | --- | --- | --- | --- | --- | --- | --- | --- |
| **Intervention Arm** | | | | **Screening** | **Eave Tubes** | **Screening** | **Eave Tubes** | **Screening** | **Eave Tubes** |
|  | Preference ranked as first choice (%) | | | 79% | 21% | 74% | 26% | 75% | 25% |
| **Installation** | | | | | | | | | |
| **All respondents** | % of  respondents for whom WTP >$0 | | Don’t know | 3% | 4% | 3% | 3% | 0% | 0% |
|  |  |  | No | 11% | 11% | 9% | 10% | 22% | 25% |
|  |  |  | Yes | 86% | 85% | 88% | 87% | 77% | 75% |
|  | Mean WTP (including zeros) | | | $8.69 | $7.62 | $8.79 | $7.67 | $ 5.79 | $ 4.75 |
|  | Mean WTP (excluding zeros) | | | $9.81 | $8.64 | $9.74 | $8.58 | $7.47 | $6.29 |
| **Screening arm** | % of respondents for whom WTP > $0 | Don’t know | | 3% | 3% | 0% | 1% | 1% | 2% |
|  |  | No | | 15% | 16% | 7% | 10% | 22% | 25% |
|  |  | Yes | | 82% | 81% | 93% | 89% | 77% | 73% |
|  | Mean WTP (including zeros) | | | $6.66 | $5.46 | $10.27 | $8.49 | $6.91 | $5.22 |
|  | Mean WTP (excluding zeros) | | | $7.92 | $6.52 | $11.08 | $9.40 | $8.86 | $7.03 |
| **Eave tubes arm** | % of respondents for whom WTP > $0 | Don’t know | | 5% | 6% | 2% | 2% | 0% | 0% |
|  |  | No | | 8% | 9% | 9% | 9% | 26% | 26% |
|  |  | Yes | | 87% | 86% | 89% | 89% | 73% | 73% |
|  | Mean WTP (including zeros) | | | $10.92 | $9.77 | $12.16 | $10.61 | $6.91 | $6.11 |
|  | Mean WTP (excluding zeros) | | | $11.98 | $10.75 | $12.18 | $11.68 | $9.40 | $8.61 |
| **Control arm** | % of respondents for whom WTP > $0 | Don’t know | | 3% | 5% | 5% | 5% | 0% | 2% |
|  |  | No | | 9% | 10% | 12% | 12% | 19% | 15% |
|  |  | Yes | | 88% | 86% | 83% | 83% | 81% | 82% |
|  | Mean WTP (including zeros) | | | $8.56 | $7.69 | $7.00 | $6.50 | $6.06 | $4.96 |
|  | Mean WTP (excluding zeros) | | | $9.45 | $8.55 | $7.99 | $7.44 | $7.46 | $6.30 |
| **Retreatment** | | | | | | | | | |
| **All respondents** | Mean WTP (including zeros) | | | NA | $4.88 | NA | $4.43 | NA | $2.92 |
|  | Mean WTP (excluding zeros) | | | NA | $5.65 | NA | $4.97 | NA | $4.00 |

## Regression analyses

### Supplementary Table S6 Willingness to pay one-way regression results

*One-way regression run only for the pre-installation survey to explore potential associations. Multivariable regression analysis was run for all timepoints. *Include respondents who were WTP nothing or something and excludes any “I don’t know” respondents*

|  |  |  | **Screening n=1,448*** | | | | **Eave Tube n=1,434*** | | | |
| --- | --- | --- | --- | --- | --- | --- | --- | --- | --- | --- |
| **Head of Household characteristics** | **Age** | **<30 years** | *reference* | | | | *reference* | | | |
|  |  | **31+ years** | *2.80* | *0.84* | *4.75* | 0.005 | *1.84* | *0.23* | *3.46* | 0.026 |
|  | **Education Level** | **Primary or lower** | *reference* | | | | *reference* | | | |
|  |  | **Secondary** | *1.77* | *0.01* | *3.53* | 0.048 | *2.08* | *0.61* | *3.55* | 0.006 |
|  |  | **Higher education** | *17.06* | *12.55* | *21.57* | <0.001 | *9.09* | *5.30* | *12.88* | <0.001 |
|  | **Gender** | **Female** | *reference* | | | | *reference* | | | |
|  |  | **Male** | *2.37* | *0.46* | *4.27* | 0.015 | *2.25* | *0.67* | *3.82* | 0.005 |
| **Household characteristics** | **National Wealth Quintiles** | **Poorest** | *reference* | | | | *reference* | | | |
|  |  | **Poor** | *2.75* | *0.01* | *5.46* | 0.049 | *2.38* | *0.13* | *4.64* | 0.039 |
|  |  | **Middle** | *3.68* | *1.11* | *6.26* | 0.005 | *3.55* | *1.42* | *5.68* | 0.001 |
|  |  | **Wealthy** | *6.73* | *3.70* | *9.76* | <0.001 | *5.88* | *3.37* | *8.39* | <0.001 |
|  |  | **Wealthiest** | *13.64* | *9.13* | *18.16* | <0.001 | *10.76* | *7.01* | *14.51* | <0.001 |
|  | **Study Arm** | **Control** | *reference* | | | | *reference* | | | |
|  |  | **Eave Tubes** | *2.36* | *0.37* | *4.35* | 0.02 | *2.08* | *0.43* | *3.72* | 0.014 |
|  |  | **Screening** | *-1.89* | *-3.88* | *0.09* | 0.061 | *-2.23* | *-3.87* | *0.60* | 0.008 |
|  | **Number of household members** | **Continuous** | *1.07* | *0.70* | *1.44* | <0.001 | *0.85* | *0.54* | *1.15* | <0.001 |
|  | **Adequate LLIN coverage** | **No** | *reference* | | | | *reference* | | | |
|  |  | **Yes** | *2.37* | *0.34* | *4.27* | 0.015 | *1.12* | *-0.31* | *2.54* | 0.125 |

### Supplementary Table S7 Two-part model for Screening WTP at three timepoints

*Our dependent variable was highly skewed with a large mass of zeros, so we used a two-part model; the first part estimated a logit model using the full sample and gave probability that the respondent’s WTP was greater than 0 and the second part estimated a generalised linear model on the subset of respondents whose WTP was greater than 0. We used a Box-Cox test to choose the natural log link function and the modified Park test to choose a Gamma distribution. To find a parsimonious model, we started by including all independent variables in the selection equation and primary equation.* *We used likelihood ratio tests to decide whether to remove a variable from the model, with each iteration removing the variable with the highest p-value. We included age as a linear variable during robustness checks and saw no difference. We performed goodness of fit tests (Pearson’s correlation, Pregibon link, and modified Hosmer-Lemeshow tests) to ensure correct model specification. *Coef.=coefficient**Model variable predicted perfectly*

1. **Pre-installation**

| **Explanatory Variables** | |  | **Two Part Model** | | | | | | | |
| --- | --- | --- | --- | --- | --- | --- | --- | --- | --- | --- |
|  |  |  | **Logit Model** | | | **Generalised Linear Model** | | | **Marginal Effects** | |
|  |  |  | **n=1400** | | | **n=1283** | | |  |  |
|  |  |  | **Odds ratio** | **p-value** | **95%CI** | **Odds ratio** | **p-value** | **95%CI** | **Coef.** | **p-value** |
| **Head of Household characteristics** | **Education Level** | **Primary or lower** | ref | | | | | | | |
|  |  | **Secondary** | 1.62 | 0.027 | (1.05 - 2.49) | 1.08 | 0.392 | (0.90 - 1.28) | 0.98 | 0.204 |
|  |  | **Higher education** | predicted perfectly** |  |  | 2.32 | <0.001 | (1.48 - 3.61) | 10.34 | 0.01 |
|  | **Gender** | **Female** | ref | | | | | | | |
|  |  | **Male** | 1.93 | <0.001 | (1.34 - 2.77) | 1.02 | 0.815 | (0.84 - 1.24) | 0.68 | 0.401 |
| **Household characteristics** | **Household wealth** | **Poorest** | ref | | | | | | | |
|  |  | **Poor** | 1.75 | 0.023 | (1.07 - 2.83) | 1.26 | 0.095 | (0.96 - 1.65) | 2.00 | 0.027 |
|  |  | **Middle** | 1.80 | 0.012 | (1.13 - 2.84) | 1.30 | 0.047 | (1.00 - 1.69) | 2.27 | 0.008 |
|  |  | **Wealthy** | 3.16 | 0.001 | (1.59 - 6.26) | 1.67 | 0.001 | (1.23 - 2.26) | 4.96 | <0.001 |
|  |  | **Wealthiest** | 4.50 | 0.047 | (1.02 - 19.8) | 1.97 | 0.003 | (1.25 - 3.08) | 7.08 | 0.007 |
|  | **Number of people in house** | **continuous** | 1.15 | 0.003 | (1.04 - 1.25) | 2.97 | <0.001 | (1.07 - 1.15) | 0.99 | <0.001 |
|  | **Study Arm** | **Control** | Ref | | | | | | | |
|  |  | **Eave Tubes** | 1.08 | 0.729 | (0.69 - 1.69) | 1.17 | 0.101 | (0.96 - 1.41) | 1.54 | 0.095 |
|  |  | **Screening** | 0.56 | 0.004 | (0.37 - 0.82) | 0.70 | <0.001 | (0.58 - 0.85) | -2.92 | <0.001 |
|  | **Adequate LLIN coverage** | **no** | Ref | | | | | | | |
|  |  | **yes** | 1.18 | <0.001 | (0.81 - 2.57) | 1.23 | 0.018 | (1.03 - 1.46) | 1.83 | 0.013 |
|  | **Pseudo R2** |  | 0.071 |  |  |  |  |  |  |  |
|  | **Deviance** |  |  |  |  | 1591.2 |  |  |  |  |
|  | **Pearson’s** |  |  |  |  | 2522.6 |  |  |  |  |
|  | **AIC** |  |  |  |  | 22.66 |  |  |  |  |
|  | **BIC** |  |  |  |  | -7505.3 |  |  |  |  |

1. **12 months post-installation**

| **Explanatory Variables** | |  | **Two Part Model** | | | | | | | |
| --- | --- | --- | --- | --- | --- | --- | --- | --- | --- | --- |
|  |  |  | **Logit Model** | | | **Generalised Linear Model** | | | **Marginal Effects** | |
|  |  |  | **n=1446** | | | **n=1271** | | |  |  |
|  |  |  | **Odds ratio** | **p-value** | **95%CI** | **Odds ratio** | **p-value** | **95%CI** | **Coef.** | **p-value** |
| **Head of Household characteristics** | **Education Level** | **Primary or lower** | ref | | | | | | | |
|  |  | **Secondary** | 1.91 | 0.003 | (1.24 - 2.93) | 1.26 | 0.009 | (1.06 - 1.50) | 2.57 | 0.003 |
|  |  | **Higher education** | 1.14 | 0.811 | (0.38 - 3.36) | 2.00 | 0.004 | (1.24 - 3.22) | 7.80 | 0.037 |
|  | **Gender** | **Female** | ref | | | | | | | |
|  |  | **Male** | 1.21 | 0.296 | (0.84 - 1.74) | 0.98 | 0.819 | (0.81 - 1.17) | -0.02 | 0.984 |
| **Household characteristics** | **Household wealth** | **Poorest** | ref | | | | | | | |
|  |  | **Poor** | 0.95 | 0.844 | (0.59 - 1.53) | 1.22 | 0.097 | (0.96 - 1.54) | 1.18 | 0.105 |
|  |  | **Middle** | 0.89 | 0.628 | (0.56 - 1.41) | 1.64 | 0 | (1.29 - 2.06) | 3.37 | 0 |
|  |  | **Wealthy** | 1.23 | 0.504 | (0.67 - 2.25) | 2.36 | 0 | (1.79 - 3.11) | 7.72 | 0 |
|  |  | **Wealthiest** | 2.85 | 0.169 | (0.64 - 12.6) | 2.24 | 0 | (1.43 - 3.50) | 7.71 | 0.005 |
|  | **Number of people in house** | **continuous** | 1.11 | 0.023 | (1.01 - 1.21) | 1.07 | 0 | (1.03 - 1.11) | 0.69 | <0.001 |
|  | **Study Arm** | **Control** | ref | | | | | | | |
|  |  | **Eave Tubes** | 1.69 | 0.007 | (1.15 - 2.48) | 1.65 | 0 | (1.36 - 1.99) | 4.60 | <0.001 |
|  |  | **Screening** | 2.79 | <0.001 | (1.83 - 4.24) | 1.46 | 0 | (1.21 - 1.75) | 3.75 | <0.001 |
|  | **Adequate LLIN coverage** | **no** | ref | | | | | | | |
|  |  | **yes** | 0.66 | 0.016 | (0.46 - 0.92) | 0.80 | 0.006 | (0.68 - 0.93) | -2.25 | 0.002 |
|  | **Pseudo R2** |  | 0.0502 |  |  |  |  |  |  |  |
|  | **Deviance** |  |  |  |  | 1329.054 |  |  |  |  |
|  | **Pearson’s** |  |  |  |  | 2407.786 |  |  |  |  |
|  | **AIC** |  |  |  |  | 22.7992 |  |  |  |  |
|  | **BIC** |  |  |  |  | -7669.72 |  |  |  |  |

1. **18-months post-installation**

| **Explanatory Variables** | |  | **Two Part Model** | | | | | | | |
| --- | --- | --- | --- | --- | --- | --- | --- | --- | --- | --- |
|  |  |  | **Logit Model** | | | **Generalised Linearized Model** | | | **Marginal Effects** | |
|  |  |  | **n=1493** | | | **n=1158** | | |  |  |
|  |  |  | **Odds ratio** | **p-value** | **95%CI** | **Odds ratio** | **p-value** | **95%CI** | **Coef.** | **p-value** |
| **Head of Household characteristics** | **Education Level** | **Primary or lower** | Ref | | | | | | | |
|  |  | **Secondary** | 0.75 | 0.046 | (0.55 - 0.99) | 1.36 | <0.001 | (1.15 - 1.59) | 1.52 | 0.009 |
|  |  | **Higher education** | 1.36 | 0.546 | (0.50 - 3.66) | 1.47 | 0.088 | (0.94 - 2.29) | 3.02 | 0.132 |
|  | **Gender** | **Female** | Ref | | | | | | | |
|  |  | **Male** | 1.80 | <0.001 | (1.36 - 2.37) | 1.04 | 0.603 | (0.88 - 1.23) | 0.99 | 0.064 |
| **Household characteristics** | **Household wealth** | **Poorest** | Ref | | | | | | | |
|  |  | **Poor** | 1.69 | 0.004 | (1.18 - 2.42) | 1.48 | 0.001 | (1.17 - 1.87) | 2.14 | <0.001 |
|  |  | **Middle** | 2.50 | <0.001 | (1.74 - 3.57) | 1.62 | <0.001 | (1.29 - 2.02) | 3.08 | <0.001 |
|  |  | **Wealthy** | 2.33 | <0.001 | (1.48 - 3.64) | 2.34 | <0.001 | (1.79 - 3.04) | 5.65 | <0.001 |
|  |  | **Wealthiest** | 1.64 | 0.129 | (0.86 - 3.10) | 3.29 | <0.001 | (2.22 - 4.86) | 8.33 | <0.001 |
|  | **Number of people in house** | **continuous** | 1.05 | 0.115 | (0.98 - 1.12) | 1.07 | <0.001 | (1.03 - 1.10) | 0.47 | <0.001 |
|  | **Study Arm** | **Control** | Ref | | | | | | | |
|  |  | **Eave Tubes** | 0.66 | 0.008 | (0.48 - 0.89) | 1.20 | 0.035 | (1.01 - 1.42) | 0.59 | 0.289 |
|  |  | **Screening** | 0.82 | 0.232 | (0.60 - 1.13) | 1.16 | 0.091 | (0.97 - 1.36) | 0.65 | 0.233 |
|  | **Adequate LLIN coverage** | **no** | Ref | | | | | | | |
|  |  | **yes** | Omitted** |  |  |  |  |  |  |  |
|  | **Pseudo R2** |  | 0.0414 |  |  |  |  |  |  |  |
|  | **Deviance** |  |  |  |  | 823.728 |  |  |  |  |
|  | **Pearson’s** |  |  |  |  | 1651.277 |  |  |  |  |
|  | **AIC** |  |  |  |  | 22.39242 |  |  |  |  |
|  | **BIC** |  |  |  |  | -7267.73 |  |  |  |  |

### Supplementary Table S8 Two-part model for Eave Tubes WTP at three timepoints

1. **Pre-installation**

| **Explanatory Variables** | |  | **Two Part Model** | | | | | | | |
| --- | --- | --- | --- | --- | --- | --- | --- | --- | --- | --- |
|  |  |  | **Logit Model** | | | **Generalised Linear Model** | | | **Marginal Effects** | |
|  |  |  | **n=1434** | | | **n=1264** | | |  |  |
|  |  |  | **Odds ratio** | **p-value** | **95%CI** | **Odds ratio** | **p-value** | **95%CI** | **Coef.** | **p-value** |
| **Head of Household characteristics** | **Education Level** | **Primary or lower** | Ref | | | | | | | |
|  |  | **Secondary** | 1.79 | 0.009 | (1.15 - 2.75) | 1.10 | 0.271 | (0.92 - 1.30) | 1.11 | 0.111 |
|  |  | **Higher education** | 4.67 | 0.134 | (0.62 - 35.2) | 1.59 | 0.035 | (1.03 - 2.45) | 5.28 | 0.05 |
|  | **Gender** | **Female** | Ref | | | | | | | |
|  |  | **Male** | 2.00 | <0.001 | (1.39 - 2.85) | 1.09 | 0.399 | (0.89 - 1.31) | 1.10 | 0.145 |
| **Household characteristics** | **Household wealth** | **Poorest** | Ref | | | | | | | |
|  |  | **Poor** | 1.53 | 0.088 | (0.93 - 2.48) | 1.31 | 0.046 | (1.00 - 1.71) | 1.89 | 0.016 |
|  |  | **Middle** | 1.60 | 0.048 | (1.00 - 2.53) | 1.39 | 0.011 | (1.08 - 1.79) | 2.36 | 0.002 |
|  |  | **Wealthy** | 2.32 | 0.011 | (1.21 - 4.44) | 1.81 | <0.001 | (1.34 - 2.42) | 4.83 | <0.001 |
|  |  | **Wealthiest** | 4.29 | 0.055 | (0.96 - 18.9) | 2.22 | <0.001 | (1.43 - 3.42) | 7.46 | 0.002 |
|  | **Number of people in house** | **continuous** | 1.16 | 0.001 | (1.05 - 1.26) | 1.10 | <0.001 | (1.06 - 1.14) | 0.86 | <0.001 |
|  | **Study Arm** | **Control** | Ref | | | | | | | |
|  |  | **Eave Tubes** | 1.14 | 0.549 | (0.73 - 1.78) | 1.14 | 0.173 | (0.94 - 1.36) | 1.23 | 0.148 |
|  |  | **Screening** | 0.59 | 0.008 | (0.39 - 0.86) | 0.67 | <0.001 | (0.55 - 0.80) | -3.02 | <0.001 |
|  | **Adequate LLIN coverage** | **no** | Ref | | | | | | | |
|  |  | **yes** | 1.16 | 0.435 | (0.79 - 1.68) | 1.31 | 0.002 | (1.10 - 1.54) | 2.15 | 0.002 |
|  | **Pseudo R2** |  | 0.0762 |  |  |  |  |  |  |  |
|  | **Deviance** |  |  |  |  | 1510.9 |  |  |  |  |
|  | **Pearson’s** |  |  |  |  | 2342.25 |  |  |  |  |
|  | **AIC** |  |  |  |  | 22.42 |  |  |  |  |
|  | **BIC** |  |  |  |  | -7430.89 |  |  |  |  |

1. **12-months post-installation-**

| **Explanatory Variables** | |  | **Two Part Model** | | | | | | | |
| --- | --- | --- | --- | --- | --- | --- | --- | --- | --- | --- |
|  |  |  | **Logit Model** | | | **Generalised Linear Model** | | | **Marginal Effects** | |
|  |  |  | **n=1441** | | | **n=1248** | | |  |  |
|  |  |  | **Odds ratio** | **p-value** | **95%CI** | **Odds ratio** | **p-value** | **95%CI** | **Coef.** | **p-value** |
| **Head of Household characteristics** | **Education Level** | **Primary or lower** | Ref | | | | | | | |
|  |  | **Secondary** | 2.30 | <0.001 | (1.48 - 3.54) | 1.30 | 0.005 | (1.08 - 1.56) | 2.66 | 0.001 |
|  |  | **Higher education** | 0.79 | 0.623 | (0.31 - 1.99) | 2.23 | 0.002 | (1.33 - 3.72) | 7.52 | 0.043 |
|  | **Gender** | **Female** | Ref | | | | | | | |
|  |  | **Male** | 1.17 | 0.387 | (0.82 - 1.65) | 0.91 | 0.34 | (0.75 - 1.10) | -0.57 | 0.446 |
| **Household characteristics** | **Household wealth** | **Poorest** | Ref | | | | | | | |
|  |  | **Poor** | 0.92 | 0.706 | (0.57 - 1.44) | 1.31 | 0.037 | (1.01 - 1.68) | 1.42 | 0.045 |
|  |  | **Middle** | 0.93 | 0.738 | (0.59 - 1.44) | 1.66 | <0.001 | (1.29 - 2.13) | 3.11 | <0.001 |
|  |  | **Wealthy** | 1.40 | 0.276 | (0.76 - 2.53) | 2.04 | <0.001 | (1.52 - 2.73) | 5.35 | <0.001 |
|  |  | **Wealthiest** | 3.27 | 0.12 | (0.73 - 14.5) | 2.34 | <0.001 | (1.46 - 3.73) | 7.49 | 0.005 |
|  | **Number of people in house** | **continuous** | 1.10 | 0.027 | (1.01 - 1.19) | 1.06 | 0.004 | (1.02 - 1.10) | 0.54 | 0.001 |
|  | **Study Arm** | **Control** | Ref | | | | | | | |
|  |  | **Eave Tubes** | 1.81 | 0.002 | (1.23 - 2.66) | 1.64 | <0.001 | (1.34 - 1.99) | 4.18 | <0.001 |
|  |  | **Screening** | 1.93 | <0.001 | (1.33 - 2.81) | 1.35 | 0.003 | (1.11 - 1.64) | 2.54 | <0.001 |
|  | **Adequate LLIN coverage** | **no** | Ref | | | | | | | |
|  |  | **yes** | 0.61 | 0.003 | (0.43 - 0.84) | 0.79 | 0.006 | (0.66 - 0.93) | -2.18 | 0.001 |
|  | **Pseudo R2** |  | 0.0489 |  |  |  |  |  |  |  |
|  | **Deviance** |  |  |  |  | 1311.516 |  |  |  |  |
|  | **Pearson’s** |  |  |  |  | 2614.1 |  |  |  |  |
|  | **AIC** |  |  |  |  | 22.586 |  |  |  |  |
|  | **BIC** |  |  |  |  | -7500.3 |  |  |  |  |

1. **18-months post-installation**

| **Explanatory Variables** | |  | **Two Part Model** | | | | | | | |
| --- | --- | --- | --- | --- | --- | --- | --- | --- | --- | --- |
|  |  |  | **Logit Model** | | | **Generalised Linear Model** | | | **Marginal Effects** | |
|  |  |  | **n=1489** | | | **n=1124** | | |  |  |
|  |  |  | **Odds ratio** | **p-value** | **95%CI** | **Odds ratio** | **P-value** | **95%CI** | **Coef.** | **p-value** |
| **Head of Household characteristics** | **Education Level** | **Primary or lower** | Ref | | | | | | | |
|  |  | **Secondary** | 0.77 | 0.07 | (0.58 - 1.02) | 1.25 | 0.006 | (1.06 - 1.47) | 0.83 | 0.074 |
|  |  | **Higher education** | 1.56 | 0.374 | (0.58 - 4.17) | 1.55 | 0.047 | (1.00 - 2.40) | 3.15 | 0.078 |
|  | **Gender** | **Female** | Ref | | | | | | | |
|  |  | **Male** | 1.63 | <0.001 | (1.24 - 2.13) | 1.07 | 0.447 | (0.90 - 1.25) | 0.86 | 0.05 |
| **Household characteristics** | **Household wealth** | **Poorest** | Ref | | | | | | | |
|  |  | **Poor** | 1.67 | 0.004 | (1.17 - 2.36) | 1.41 | 0.004 | (1.11 - 1.78) | 1.66 | <0.001 |
|  |  | **Middle** | 2.56 | <0.001 | (1.80 - 3.63) | 1.53 | <0.001 | (1.22 - 1.90) | 2.44 | <0.001 |
|  |  | **Wealthy** | 1.97 | 0.002 | (1.28 - 3.00) | 2.24 | <0.001 | (1.71 - 2.90) | 4.40 | <0.001 |
|  |  | **Wealthiest** | 1.84 | 0.065 | (0.96 - 3.50) | 3.07 | <0.001 | (2.07 - 4.53) | 6.83 | <0.001 |
|  | **Number of people in house** | **continuous** | 1.04 | 0.233 | (0.97 - 1.10) | 1.06 | 0.001 | (1.02 - 1.09) | 0.32 | 0.001 |
|  | **Study Arm** | **Control** | Ref | | | | | | | |
|  |  | **Eave Tubes** | 0.77 | 0.086 | (0.57 - 1.03) | 1.28 | 0.004 | (1.08 - 1.51) | 0.96 | 0.041 |
|  |  | **Screening** | 0.77 | 0.095 | (0.57 - 1.04) | 1.11 | 0.246 | (0.93 - 1.30) | 0.21 | 0.632 |
|  | **Adequate LLIN coverage** | **no** | Ref | | | | | | | |
|  |  | **yes** | Omitted |  |  |  |  |  |  |  |
|  | **Pseudo R2** |  | 0.0333 |  |  |  |  |  |  |  |
|  | **Deviance** |  |  |  |  | 764.5 |  |  |  |  |
|  | **Pearson’s** |  |  |  |  | 1537.3 |  |  |  |  |
|  | **AIC** |  |  |  |  | 22.068 |  |  |  |  |
|  | **BIC** |  |  |  |  | -7053.92 |  |  |  |  |

## Elasticities

### Supplementary Table S9 Point elasticities for housing modifications

| **Intervention** | **Screening** | | | **Eave Tubes** | | |
| --- | --- | --- | --- | --- | --- | --- |
| **Survey Timepoint** | **Pre-installation** | **12-month** | **18-month** | **Pre-installation** | **12-month** | **18-month** |
| **All** | 0.476 | 0.409 | 0.356 | 0.464 | 0.398 | 0.343 |
| **Study Arm** |  |  |  |  |  |  |
| Control | 0.467 | 0.376 | 0.338 | 0.469 | 0.368 | 0.315 |
| Screening Arm | 0.487 | 0.410 | 0.378 | 0.459 | 0.402 | 0.353 |
| Eave Tubes arm | 0.463 | 0.439 | 0.365 | 0.452 | 0.421 | 0.367 |
| **National Wealth Quintile** | |  |  |  |  |  |
| Poorest | 0.479 | 0.349 | 0.376 | 0.463 | 0.333 | 0.388 |
| Poor | 0.468 | 0.382 | 0.311 | 0.463 | 0.386 | 0.298 |
| Middle | 0.482 | 0.414 | 0.314 | 0.462 | 0.404 | 0.301 |
| Wealthy | 0.452 | 0.440 | 0.411 | 0.457 | 0.402 | 0.387 |
| Wealthiest | 0.493 | 0.460 | 0.414 | 0.489 | 0.461 | 0.436 |

# Organic uptake outside study

### Supplementary Table S10 Construction Worker Survey Results

*All costs in constant 2022 USD.*

| **Characteristics of workers** | **Response** | **Observations (%)** |
| --- | --- | --- |
| Age | Mean (range) | 36 (18-67) |
| Community | Within study area | 48 (41%) |
|  | Within pilot area | 8 (12%) |
|  | Neighbouring village | 18 (16%) |
|  | Far away | 42 (36%) |
| Type of construction worker | Carpenter | 74 (64%) |
|  | Mason | 30 (26%) |
|  | Porter | 10 (9%) |
|  | Foreman | 2 (2%) |
| Length in profession | 1-5 years | 39 (34%) |
|  | 5+ years | 77 (66%) |
| **Pre-study modifications** |  |  |
| Before the study, how many homes have you installed screens in? | None | 72 (62%) |
|  | 1-5 homes | 28 (24%) |
|  | 6-10 homes | 7 (6%) |
|  | 10+ homes | 9 (8%) |
| What did you charge per window? | Mean (range) | $25 ($6 - $141) |
| **Post-study modifications** |  |  |
| Since study completed, how many people have asked you to install screening at their homes? | None | 44 (38%) |
|  | 1-5 people | 47 (41%) |
|  | 6-10 people | 9 (8%) |
|  | 9+ | 15 (13%) |
| How many homes have you installed screening in? | 0 | 72 (63%) |
|  | 1 | 14 (12%) |
|  | 2 | 12 (10%) |
|  | 3+ | 17 (15%) |
| Total number of homes installed screening by construction works since study | 166 | |
| In the houses in which you installed screening, did you screen all windows or only some? | All | 29 (67%) |
|  | Some | 14 (33%) |
| Did you charge by the window or house? | Window | 12 (27%) |
|  | House | 31 (71%) |
| What did you charge per house? | Mean (range) | $ 123 ($6 - $1,211) |
| What did you charge per window? | Mean (range) | $ 22 ($3 - $168) |
| What did you charge per vent? | Mean (range) | $ 5 ($1 - $11) |
| **Hypothetical Estimations** |  |  |
| What would you charge per small window? | Mean (range) | $ 15 ($3 - $70) |
| What would you charge per medium window? | Mean (range) | $ 23 ($3 - $113) |
| What would you charge per large window? | Mean (range) | $ 31 ($3 - $239) |
| What do you think people in your community will pay to screen their homes? | Mean (range) | $ 59 (48 - 70) |
| What would you charge to install an eave tube? (excluding the cost of the PVC pop and mesh inserts) | Mean (range) | $ 7 ($1 - $56) |
